# Consumer Acceptance of Sustainable Cat Diets: A Survey of 1380 Cat Guardians

**DOI:** 10.3390/ani15202984

**Published:** 2025-10-15

**Authors:** Jenny L. Mace, Alexander Bauer, Andrew Knight, Billy Nicholles

**Affiliations:** 1 Centre for Ethics, Philosophy and Public Affairs, University of St Andrews, St Andrews, Fife KY16 9AL, UK; maceanimalwelfare@gmail.com; 2 Sustainable Pet Food Foundation, 147 Station Rd, London E4 6AG, UK; baueralexander@posteo.de (A.B.); billy@bryantresearch.co.uk (B.N.); 3 Mace Animal Welfare, Dunnock House, 63 Dunnock Road, Dunfermline, Fife KY11 8QE, UK; 4 School of Veterinary Medicine, College of Environmental and Life Sciences, Murdoch University, 90 South St., Murdoch, WA 6150, Australia; 5 School of Environment and Science, Griffith University, Nathan, QLD 4111, Australia; 6 Animal Welfare Research Group, University of Winchester, Sparkford Road, Winchester SO22 4NR, UK; 7 Bryant Research, 71–75 Shelton Street, Covent Garden, London WC2H 9JQ, UK

**Keywords:** cat food, vegan, environment, sustainability, pet food, pet diet

## Abstract

**Simple Summary:**

There are a multitude of pet diets available for the world’s 476 million pet cats. Among these, an increasing range of sustainable cat foods aims to tackle the environmental, ethical, and other challenges associated with meat-based cat food. This survey of 1380 cat guardians sought to better understand the factors guiding their food purchasing decisions—concerning both current diets, and sustainable alternatives. We found that 51% of cat guardians currently feeding conventional or raw meat-based cat food felt that at least one sustainable alternative was acceptable. Cultivated meat-based cat food was the most popular alternative, followed by nutritionally sound vegan cat food. Guardians reported that the most important feature influencing their willingness to purchase these alternatives was good health outcomes. The dietary choices of the cat guardian were strongly associated with the dietary habits of their cats. Regarding information sources, labels/packaging and veterinarians were most used, with the possible exception of guardians feeding unconventional diets, who appeared to use veterinary advice less often. Since respondents were disproportionately female, from the UK, highly educated, and feeding vegan diets, reported relative frequencies were not fully representative. To minimize any resultant bias effects, we used regression analyses for the calculation of all association estimates.

**Abstract:**

There is increasing awareness about the adverse environmental and ‘food’ animal welfare impacts associated with the production of meat-based pet food. However, little is known about cat guardians’ acceptance of more sustainable food choices for the global population of approximately 476 million pet cats. By surveying 1380 cat guardians, this study explored feeding patterns used by guardians, determinants of their cat food choices, and their acceptance levels of more sustainable cat food alternatives. The sources of information used by cat guardians to obtain information about the cat diets they chose were also investigated. Key results included: (1) 51% (620/1211) of cat guardians currently feeding meat-based cat food (raw or conventional) considered at least one or more sustainable alternatives to be acceptable, with cultivated meat-based cat food being the most popular alternative, followed by nutritionally sound vegan cat food; (2) the top five characteristics alternative diets needed to offer to be considered viable were good health outcomes, nutritional soundness, palatability, quality, and environmental sustainability; (3) diet types consumed by cat guardians and their cats were strongly associated; and (4) labels/packaging and veterinarians were the information sources most used, although veterinary staff may have been less trusted as reliable sources of dietary advice by guardians feeding unconventional diets. It should be noted that, due to the reliance on convenience sampling and the overrepresentation of respondents from the UK, of female guardians, of respondents with higher education and of vegan guardians, the reported relative frequencies of subgroups were not fully representative of the global cat guardian population. Association estimates were based on regression analyses to minimize any resultant bias effects.

## 1. Introduction

Cats rank second, after dogs, in terms of worldwide pet popularity with estimates suggesting the global pet cat population as of 2024 to be around 476 million relative to 528 million pet dogs [1]. However, cats rank first in some regions such as Europe [2]. The number of households with a cat continues to rise globally. For instance, across Europe, between 2010 and 2022, there was nearly a 50% increase in the number of households with a cat [3], and China’s pet cat population grew by over 18% annually between 2018 and 2022, with a pet cat population of 60 million expected by 2025 [4]. This is because of a growing global middle class (increasing disposable income), global events such as COVID-19, increased awareness surrounding the benefits for people arising from the human–animal bond, and continued human population growth [5]. These figures have resulted in a global cat food market worth over USD 31 billion and a forecasted annual growth rate of 4.4% up to 2030 [6]. However, concerns have been raised about the effects of conventional cat food on (1) cat health, (2) the environment, and (3) the welfare of intensively farmed and wild-caught animals used for meat production.

First, some health concerns center around perceptions of poor quality cat food. This stems from the use of animal by-products (ABPs) from slaughterhouses within cat food [7], from the potential presence of hazardous levels of toxins [8], and from frequent product recalls [9]. Some are also concerned about ultra-high processing of cat food, which can degrade many nutrients [10] (p. 6). Second, through the support of industrialized animal agriculture, conventional cat food contributes significantly to environmental strains. This is due to industrialized animal farming’s well-documented, very large contributions to deforestation and other habitat destruction, water pollution, high land and other resource use, and climate change [11,12,13]. Indeed, Okin [14] calculated, through predominately although not exclusively conservative assumptions, that US companion cats and dogs account for at least a quarter of all environmental impacts stemming from industrialized animal farming (separate calculations for dogs and cats were not provided).

Third, feeding pet cats animal-based pet food depends on the killing of another animal—unless cultivated (i.e., lab-grown) pet food is used, which is discussed in the next section. Evidence suggests that pet guardians care about all types of animals and have a higher concern for animal welfare than the general population [15]. There are also a higher number of vegetarians and vegans among pet guardians than within general populations [16]. Thus, there is a moral inconsistency and discomfort in ‘doting’ on one group of animals, while supporting the killing of another group of animals—a phenomenon Rothgerber [17] named *the vegetarian’s dilemma*, and Milburn [18], *the animal lover’s paradox*. Attempts to resolve this frequently rely on the argument that conventional pet food uses only ABPs from slaughterhouses that are unsuitable for humans, and the perspective that this removes any welfare or environmental concerns [19]. However, this view has been challenged; in a prior study we showed that almost 50% (49.2%) of animal-based cat food ingredients in the USA are suitable for human consumption, and that environmental impacts are increased, rather than decreased, through use of the ABPs which comprise the remainder of the animal-based ingredients used [12].

There are also reports of possible shortages of cat food if reliance on slaughterhouse ABPs continues, due to competing demands for ABPs for use in renewable energy production [20] concurrently with rising cat numbers and increasing cat food needs. Stimulated by the increase in such animal health/welfare, environmental, and ethical concerns among pet guardians, as well as concerns about future security of the ingredient supply chain as demand and competition increases, there are a growing number of alternative cat food options now available or under development.

### 1.1. Sustainable Alternatives to Meat-Based Cat Food

Existing or forthcoming more sustainable alternatives to meat-based (conventional or raw) cat food include those based on ingredients such as plants, algae, fungi, cultivated (i.e., laboratory-grown or cell-based) meat, proteins from fermented microorganisms, and potentially insects. Nutritionally sound vegan cat food (i.e., excluding any animal products), for example, is normally based on plants, as well as mineral and synthetic supplements, but could also include algae, fungi, and microbial proteins (although not cultivated meat). Commercial, nutritionally complete vegan cat diets are already being produced by various pet food companies, such as Ami (https://www.amipetfood.com/en/products/categories/cats/ami-cats, accessed on 3 october 2025), Benevo (https://www.benevo.com/vegan-cat-food-from-benevo/, accessed on 3 october 2025), and Wild Earth (https://wildearth.com/pages/wet-cat-food, accessed on 3 october 2025). These companies use a range of plant-based ingredients such as corn, soya, and lentils, as well nutritional additives including taurine and vitamin A in their formulations. Stakeholders often include insect-based cat food among the more sustainable alternatives; however, as there is mixed evidence regarding insect sentience (e.g., see Lambert et al. [21]), this does not alleviate welfare and ethical concerns related to the use of farmed animals. Additionally, while some scholars have praised insect farming’s relatively low feed-conversion ratio or waste valorisation potential [22], the purported environmental benefits of insect farming have been significantly critiqued (e.g., Biteau et al. [23]), and arable farming is typically less burdensome on the environment [24,25].

Most of the other alternative cat foods listed above were not available or minimally available by early 2025. The cultivated meat-based cat food market had not emerged at the time of writing, but was reportedly imminent [26]. Cat diets based on proteins from fermented microorganisms were also not yet commercially available. In contrast, nutritionally sound vegan cat food was relatively widely available. The vegan cat food market was already valued at over USD 9 billion in 2023 with a forecasted value of over USD 17 billion by 2033, and an annual growth rate of 6% [27]. Numerous companies already provide nutritionally sound vegan cat foods (e.g., see https://sustainablepetfood.info/suppliers, accessed 3 october 2025) with North America dominating the market, and most growth expected in the Asia-Pacific region [28].

There is a growing body of evidence suggesting the healthfulness of nutritionally sound vegan cat food. While domesticated cats are classified as obligate carnivores—meaning they require nutrients that, in their natural environments, came from prey animals—commercial nutritionally sound vegan cat diets supplement these nutrients from synthetic, non-animal sources. By September 2025, three studies had shown equivalent or superior health outcomes for cats maintained on vegan [29,30] or almost vegan [31] diets, provided these are formulated to be nutritionally sound, as modern commercial vegan pet diets normally are [32]. Cats were typically fed these diets for at least one year—a long-term period for cats, whose average lifespan is 13–20 years. In the study by Dodd et al. [30], cats had been fed their current diet for a mean of 3.8 years. No credible published studies had provided contrary results. Additionally, Domínguez-Oliva et al. [33] conducted a systematic review of six studies examining the health impacts of vegan diets for cats. The authors recognized that no adverse effects of the diets had been found to date—and potentially some benefits. Industry bodies are beginning to formulate statements regarding how to safely transition cats to, and maintain cats on, nutritionally sound vegan cat food. For instance, the UK Pet Food Manufacturers’ Association produced a fact and guidance sheet about nutritionally sound vegan diets for cats and dogs [34]. Accordingly, the main alternative sustainable cat diet type focused on in this study was nutritionally sound vegan cat diets.

Current scientific studies exploring cat feeding patterns and cat food purchasing determinants (e.g., refs. [16,35,36,37,38]), have provided limited information about the extent to which cat guardians would realistically be willing to consider more sustainable cat diets, and the characteristics those diets would need to offer to be chosen. Such information would help the pet food industry cater for the varying needs and preferences of different pet cat guardian demographic groups and would inform veterinarians seeking to support cat guardians. Accordingly, this study was designed to explore such factors.

### 1.2. Research Questions

We explored three broad topics: (1) existing feeding patterns and purchasing determinants among cat guardians; (2) acceptance by cat guardians of more sustainable cat diets; and (3) sources used by cat guardians to obtain information about cat diets. As mentioned, among the more sustainable cat diets considered, nutritionally sound vegan diets were the only alternative relatively widely available by late 2025; the study thus focuses particularly on nutritionally sound vegan diets as a more sustainable cat food alternative. We analyzed these topics using the following specific research questions (RQs):

(1) Current diets
  - What feeding patterns exist among cat guardians?
  - What factors do cat guardians find important when choosing cat diets?
(2) Alternative diets
  - What proportion of cat guardians currently feeding meat-based (conventional or raw) cat food would realistically be willing to choose more sustainable alternatives?
  - For those willing, what characteristics would the alternative diets need to provide in order to be chosen?
(3) Information sources

Where do cat guardians source information about cat diets from?

We also sought to discover any significant associations between these RQs and a range of human and cat demographic variables. As discussed, of the more sustainable alternatives available, the vegan pet food market is by far the most developed; thus, we were particularly interested in vegan cat food as an alternative to conventional meat-based cat food.

## 2. Materials and Methods

### 2.1. Survey Design and Distribution

**Figure 1:**
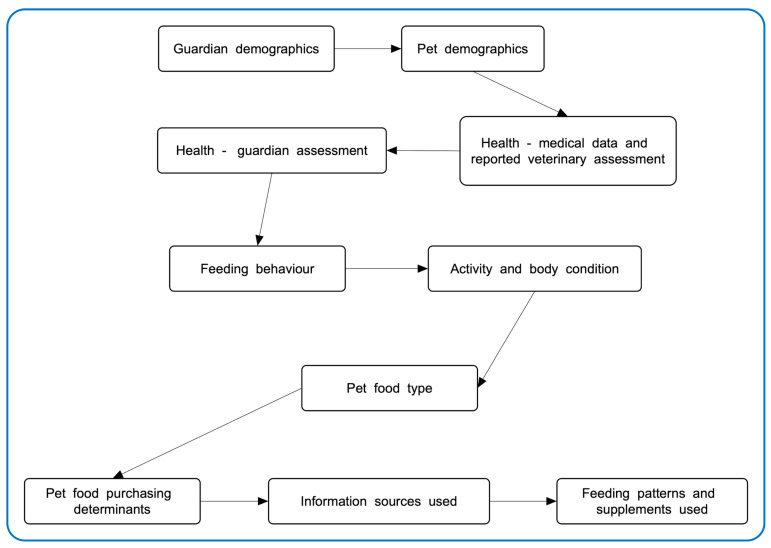
Sections of the questionnaire [39]. Note: The four sections connected to health, body condition, and behavior are not relevant to this study and so are excluded from this analysis; see Knight et al. [29,39,40] and Knight and Satchell [41] for analysis of these results.

Between May and December 2020, using a quantitative questionnaire hosted on the Online Surveys platform (https://www.onlinesurveys.ac.uk, accessed 3 october 2025), cat guardians internationally were surveyed about their cat feeding patterns, cat food purchasing determinants, acceptance of more sustainable cat food alternatives, and information sources used when making cat food diet decisions. When asking about acceptance of more sustainable cat food alternatives, the following phrasing was used: “If you could choose other options that offered your desired attributes and met your standards, might you realistically choose any of the following…” and “Many factors are desirable. But what would be essential for this new diet to provide, before you would choose it?” Thus, emphasis was placed on realistic choices in everyday life versus idealistic choices. Through this wording, the intention was to minimize any tendency in respondents to overestimate the extent to which they accept and would purchase more sustainable alternative cat food. Following a pilot survey comprising 25 respondents, edits were primarily made to the order of the survey questions. To minimize possibilities of unconscious bias, questions relating to potentially dependent variables were positioned before those relating to independent variables (aside from demographic questions, which were positioned first). This removed the risk, for instance, that answers about pet diet decisions (e.g., use of an unconventional diet) would unconsciously influence answers to other questions (e.g., about cat health status). The final survey comprised 37 key questions split into 10 sections as per Figure 1. Some respondents received additional questions depending on how they answered certain questions. Most questions were based on multiple choice options, and many allowed the selection of multiple answer options. This produced largely nominal data. The full survey is available at https://osf.io/nbepu (accessed 3 october 2025).

The survey was distributed on social media platforms, including within cat interest groups. Financed Facebook advertisements were used to promote the survey and volunteers helped to share the survey. There was some targeting of specific cat diet groups in order to achieve sufficient numbers in these groups for statistical analyses. Online Surveys was used partly because, in 2019, over 88% of UK universities used this platform [42], including our University of Winchester. Guardians were asked to consider a single cat (if they had more than one) when answering the questions, and to base their answers on experiences during the previous year. Those caring for cats on a medical diet were asked to consider their habits over the year prior to commencement of their cat’s medical diet.

### 2.2. Statistical Analysis

Respondents not agreeing to the first screening question (n = 3) were removed from the data. The cat-based data were extracted; for a similar analysis of the dog data, see Mace et al. [43]. The four sections connected to pet health and behavior were not relevant to the focus of this study and so were excluded from this analysis; see Knight et al. [29,39,40] and Knight and Satchell [41] for analysis of these results.

Inferential statistics were utilized to analyze potential differences between both human and cat demographic subgroups and RQs 1–3. Each research question was analyzed based on the estimation of multiple (generalized) linear regression models [44]. Linear regression models were estimated for (quasi-)metric response variables. Logistic regression models were estimated for binary response variables, as linear models are not suitable for such data [44]. All of the utilized category scores, outlined below, were created based on an intuitive grouping of the respective items.

For RQ1a, two logistic models were estimated on the binary questions “is the cat fed vegan or meat-based food?” and “is the cat fed raw meat or a conventional meat-based diet?” The former model was only estimated on the subgroup of vegan cat guardians because 122 out of 126 respondents feeding vegan cat diets were vegan themselves. The latter model results should be considered explorative only due to relatively few cats being fed raw meat diets (n = 60).

For RQ1b, four linear models were estimated on four quasi-metric category scores, each reflecting the share of individual items that each respondent stated were purchasing determinants for their current cat diet. The categories comprised Pet Focus I (with individual items “health and nutrition,” “palatability,” “diet quality”, i.e., pet-focused items directly influencing pet welfare), Pet Focus II (items “naturalness,” “freshness,” “diet reputation”, i.e., pet-focused items not directly influencing pet welfare), Personal Focus (items “price,” “convenience,” “social/cultural”), and Personal Values (items “food animals,” “sustainability”). For RQ2a, six logistic models were estimated on the individual items “vegan,” “fungi-based,” “algae-based,” “vegetarian,” “cultivated meat,” and “insect-based.”

For both RQ2b and RQ3, four logistic models were estimated on four category scores, each reflecting the information if at least one of the respective items was selected. The categories for RQ2b comprised Pet Focus I (items “health,” “palatability,” “quality,” “nutritional soundness”), Pet Focus II (items “naturalness,” “freshness,” “reputation”), Personal Focus (items “price,” “convenience,” “social/cultural”), and Personal Values (items “sustainability,” “animal welfare for cultivated meat,” “animal rights for cultivated meat”). For RQ3, the categories comprised Product-Specific (items “label/packaging,” “company webpage”), Vet/Pet Care (items “veterinarians,” “other vet clinic staff,” “pet store staff,” “pet paraprofessionals”), Media/Literature (items “scientific literature,” “media reports,” “non-company webpage,” “other books”), and Social Media (items “special interest group online,” “general social media”).

All models controlled for the following sets of independent variables, with the reference characteristics indicated. Categories with a sufficient number of respondents and/or positioned at the start or end of ordinal variable categories were chosen as reference characteristics. Human demographic variables comprised the respondent’s dietary category (reference “omnivore”), categorized age (“18–29”), gender (“female”), education (“doctorate”), potential occupation in the pet or veterinary industry (“no”), income level (“low”), geographical region (“UK”), and type of residence (“urban”). Cat demographic variables comprised the cat’s diet (“conventional meat-based”), whether the cat was currently fed a medical diet (“no”), age (“0–4 years”), sex and neuter status (“female, spayed”), and habitat (“mostly indoor”). As stated above, the human and cat diet effects were not estimated for all RQ1a models due to the high number of vegan-fed cats with vegan guardians.

Human diet and cat diet were both substantially correlated with each other and with the other human demographic independent variables. To prevent this “multicollinearity” from negatively affecting model estimations, the effects of human diet and cat diet were each estimated in separate regression models, which exclusively controlled for the additional cat characteristics. The resulting human and cat diet estimates are always reported side-by-side with all other estimates but are highlighted through gray-shaded areas in all figures to reflect this separated estimation scheme.

To account for the multitude of individual tests, multiple testing correction after Bonferroni-Holm [45] was applied individually for every model. Model assumptions were visually checked based on the distribution of model residuals. No relevant deviations from the assumptions could be observed. Additionally, we calculated the area under the curve (AUC) values for logistic regression models, which were calculated on a randomly selected 20% hold-out test set after re-estimating each model on the remaining 80% training set. The AUC values for RQ1a were 0.66 for the “vegan vs. meat-based” model and 0.44 for the “conventional vs. raw meat” model. The latter model should be viewed as an explorative model as only 60 cats were fed a raw meat-based diet. Other AUC values ranged between 0.57 and 0.75 (RQ2a), 0.62 to 0.71 (RQ2b), and 0.53 to 0.68 (RQ3). The R^2^ values for the RQ1b linear regression models ranged between 0.04 and 0.12. AUC values below 0.7 are generally considered to indicate poor discrimination [46]. No such hard threshold is established for R^2^ values [47]. While some of our AUC and R^2^ values were very low, this was anticipated given our focus on modeling complex personal values and interests based almost exclusively on sociodemographic factors. In such social science research settings, low goodness-of-fit values do not necessarily indicate an uninterpretable model, but observed effect patterns and significances can and should still be interpreted [47].

Microsoft Excel was used to supply descriptive statistics. Inferential analyses were conducted using the open-source statistical software R version 4.5.0 [48]. Regression models were estimated with function “gam” from package “mgcv” version 1.9-1 [49]. AUC values were calculated with function “calc_auc” from package “plotROC” version 2.3.3 [50].

Our research complied with the University of Winchester Ethics Policy [51] with the approval reference RKEEC200304_Knight. Before being granted access to the survey, respondents were informed about the purpose of the study and asked to confirm that they were 18 or over and that they consented to participate. They were also asked to confirm that they would answer the questions about one dog or cat in their care for at least a year. Participants were required to select ‘Yes’ before gaining access to the survey. Our data, along with the questionnaire, the mapping between dependent variables and questionnaire items, and the R code used for its statistical analysis, are available at https://osf.io/nbepu (accessed 3 october 2025).

## 3. Results

Data cleaning included the removal of those stating they played no role in pet food decisions (n = 17) and of those leaving all questions blank (missing data) after the demographic questions (n = 21). Following this, 1380 respondents remained. Not all respondents answered every question; thus the total number of respondents is stated for each data point. Additionally, for the regression-based analyses of associations with human and cat demographic characteristics, pregnant (n = 1) and lactating (n = 3) cats were excluded, due to their non-standard nutritional requirements. The following results describe the human and cat demographic characteristics of the remaining participants, before proceeding to explore the three key topics outlined in Section 1.2. Each subsection comprises descriptive statistics followed by the most important results from regression modeling of the associations between human/cat demographic characteristics and the variables of interest.

In reporting these results, we have applied these conventions:

- Effect = significant association after multiple testing correction.
- Trend = significant association prior to multiple testing correction.
- No trend = not significant prior to multiple testing correction.
- Explorative tendency = an association arising from explorative analyses only.
- Tendency = general pattern.

Only the most important results (effects and trends of particular interest) are outlined below; exhaustive results are supplied within Tables S1–S3.

### 3.1. Human and Cat Demographic Characteristics

Table 1 provides demographic characteristics for the 1380 cat guardians included. In summary, 89.4% (1234/1380) of respondents were female; 70.5% (973/1380) were from the UK; 75.6% (1043/1380) had some experience of university-level education; and 60.7% (837/1380) had a “medium” level of income relative to “low” (20.7%, 286/1380), “high” (9.8%, 135/1380), and “prefer not to answer” (8.8%, 122/1380). There was a fairly even spread between the ages of 20 and 69, with the most represented age range being 50–59 (20.6%, 284/1380). Similarly, 47.8% (659/1380) lived in an urban environment, but just over a quarter lived in both rural and equally urban/rural areas (26.2%, 361/1380; 25.1%, 347/1380, respectively). Moreover, 35.1% (484/1380) had a standard omnivorous diet. Additionally, 90.1% of respondents (1243/1380) did not work in the vet/pet industry, whether as a veterinarian, veterinary nurse/technician, animal trainer, or an animal breeder.

**Figure 2:**
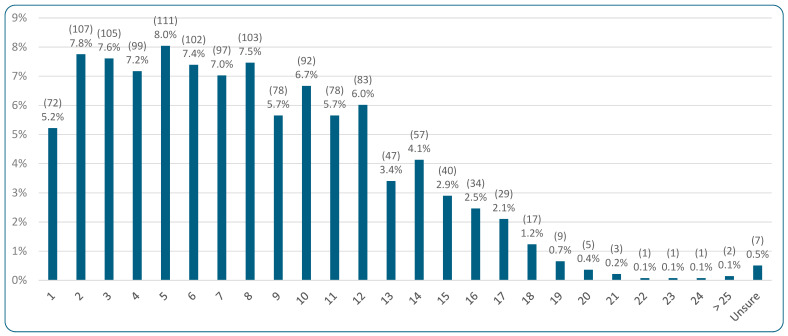
Distribution of cat ages (n = 1380).

Considering the demographic characteristics for the 1380 cats included, 99.9% (1379/1380) were regarded by their guardians as a companion rather than a working animal. The ages of these cats are indicated in Figure 2. A wide range of ages was evident, with a median age of seven years. The vast majority of cats were neutered (97.2%, 1342/1380), with figures being comparable for both female (97.2%, 689/709) and male (97.5%, 653/670) cats, and 0.1% (1/1380) unsure. Almost all cats (98.9%, 1365/1380) did not have specific characteristics (high levels of exercise, lactating, or pregnant) that could increase metabolic energy demands. Additionally, 92.1% (1271/1380) were not fed a medical (therapeutic or prescription) diet; indeed, 94.5% (n = 1305) of respondents regarded their cat as either “healthy” (62.5%, 863/1380) or “generally healthy” (32.0%, 442/1380). (Health outcomes were previously analyzed in detail by Knight et al. (2023) [29]). Over half (57.8%, 797/1380) of the cats were primarily indoor cats, while 38.5% (531/1380) spent significant amounts of time both indoors and outdoors. Just 3.7% (51/1380) spent most of their time outdoors and one guardian was unsure of their cat’s primary location. Table 2 illustrates the diets primarily fed to cats, with “meat-based—conventional” comprising the majority, at 84.2% (1162/1380).

### 3.2. Current Feeding Patterns and Purchasing Determinants

#### 3.2.1. Current Feeding Patterns

**Figure 3:**
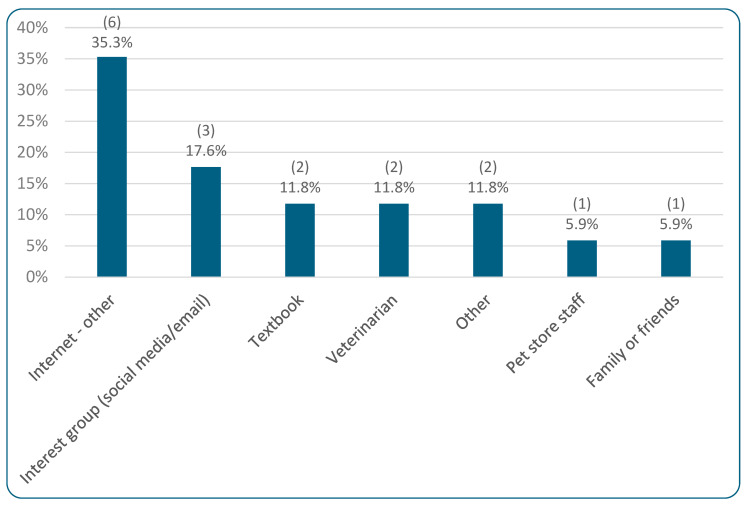
Distribution of the sources of homemade cat food recipes (n = 17).

Table 3 demonstrates where guardians obtained the majority of their cat food, with “other store (e.g., supermarket, grocery store, farmer’s market, pharmacy)” being selected most commonly by 46.2% (637/1380). Table 3 further demonstrates that commercial feed comprised 100% of the diet of 42.8% (590/1380) of respondents’ cats. Respondents were also specifically asked whether homemade food comprised more than half of their cats’ diets; 4.1% (57/1380) said “yes.” Of these 57 respondents, less than half (29.8%, 17/57) used a recipe, while 70.2% (40/57) did *not* use a recipe. Figure 3 illustrates the sources for the recipes used, with “internet” being the most common (selected by 35.3%, 6/17).

**Figure 4:**
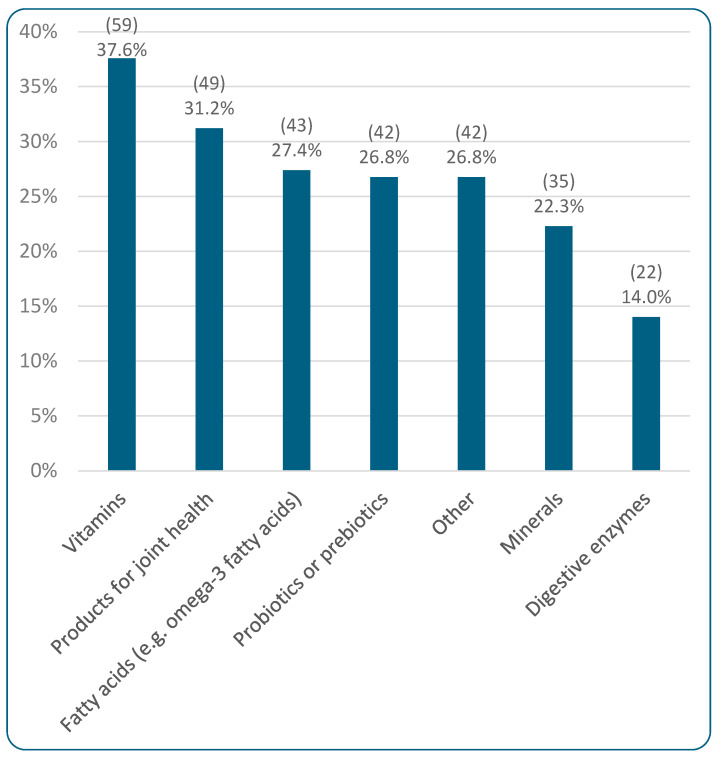
Distribution of dietary supplements given to cats. Note: Multiple responses were possible; thus, the frequencies and percentages represent the proportion of all respondents (n = 157) selecting each answer option.

“Food is always available” (36.6%, 505/1380) was the most common response to the question about feeding frequency, followed by “twice daily” (35.1%, 485/1380), “three times daily” (21.0%, 290/1380), “other” (4.3%, 60/1380), and “once daily” (2.9%, 40/1380). Common “other” responses included: when required/asked for, a mix (e.g., two wet meals per day, but kibble also always available), and 4–5 small servings. Over 80% (82.5%, 1139/1380) did not weigh the food given. Additionally, 82.8% (1142/1380) stated their cats did not compete with others for food at mealtimes, while 15.4% (212/1380) did and 1.9% (26/1380) were unsure. Nearly three quarters (74.9%, 1034/1380) fed treats/snacks/scraps to their cat as illustrated in Table 4. These were most commonly provided “once a day” (38.1%, 394/1034). The most common treat given was “other commercial treat” (68.5%, 708/1034). Over 85% (86.3%, 1191/1380) did not give their cat any supplements. Of the 13.7% (189/1,380) who did give supplements, 21.7% (41/189) gave amino acids or supplements designed to supply these, which commonly included Vegecat, taurine, carnitine, and lysine. The other supplements given are summarized in Figure 4.

**Figure 5:**
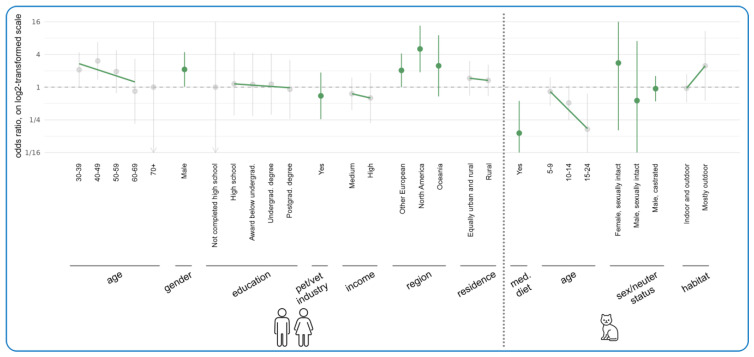
Logistic regression results on the associations between human/cat demographic characteristics and the likelihood of *currently* feeding vegan diets to cats, relative to the reference characteristics for each group. Note: Vegan cat diets were almost exclusively (96.8%) fed by people who were vegan themselves. As this association was so strong, this regression analysis was run using only vegan respondents. The reference characteristics for these humans and cats were, respectively: (human) aged 18–29, female, doctoral degree, not in pet/vet industry, low income, UK- and urban-based; (cat) no medical diet, aged 0–4, female, spayed, mostly indoors. Effects are depicted as odds ratios, including 95% confidence intervals (not corrected for multiple testing). No effect was significant after correction for multiple testing. Individual estimated effects of ordinal variables are grayed out in favor of an additional linear trend line.

Figure 5 illustrates the results of regression modeling of the associations between human/cat demographic characteristics and the likelihood of *currently* feeding vegan diets to cats. This model was limited to vegan guardians because there were only four nonvegan guardians feeding vegan cat food. The most noteworthy tendencies concerning human demographics related to age, gender, and region. As age increased, there appeared to be a decreasing tendency to feed vegan diets. However, ages 30–59 were still more likely than the reference category (aged 18–29) to feed vegan diets. This was most marked for the 40–49 age band (OR = +204%, CI: [+37%, +575%], *p* = 0.2371), which demonstrated a 204% higher chance of feeding a vegan instead of a conventional meat-based diet, compared to the reference age group (aged 18–29). For this age group (only), this constituted a trend.

Men had a 112% higher probability of feeding a vegan instead of a conventional meat-based cat diet, compared to women as the reference category (OR = +112%, CI: [+2%, +341%], *p* > 0.9999). This constituted a trend. North Americans also had a 405% higher chance of feeding a vegan instead of conventional meat-based cat diet, compared to UK residents as the reference category (OR = +405%, CI: [+88%, +1256%], *p* = 0.0519). This constituted a trend as well. Age, gender, and region effects were not significant after performing multiple-testing corrections.

When considering cat demographic characteristics, increasing cat age tended to reduce the likelihood of feeding vegan cat food; for instance, cats aged 15–24 had an 83% lower chance of being fed a vegan instead of a conventional meat-based diet, compared to cats with the reference age of 0–4 years (OR = −83%, CI: [−96%, −24%], *p* = 0.7099). This constituted a trend. Additionally, cats progressing onto a medical diet had an 86% lower chance of being fed a vegan instead of meat-based diet (OR = −86%, CI: [−96%, −44%], *p* = 0.1988). This also constituted a trend. Neither of these age/medical diet effects were significant after performing multiple-testing correction. For regression analysis results of the effect of demographic factors on the likelihood of cats being fed raw meat, see Figure S1.

#### 3.2.2. Current Diet Purchasing Determinants

**Figure 6:**
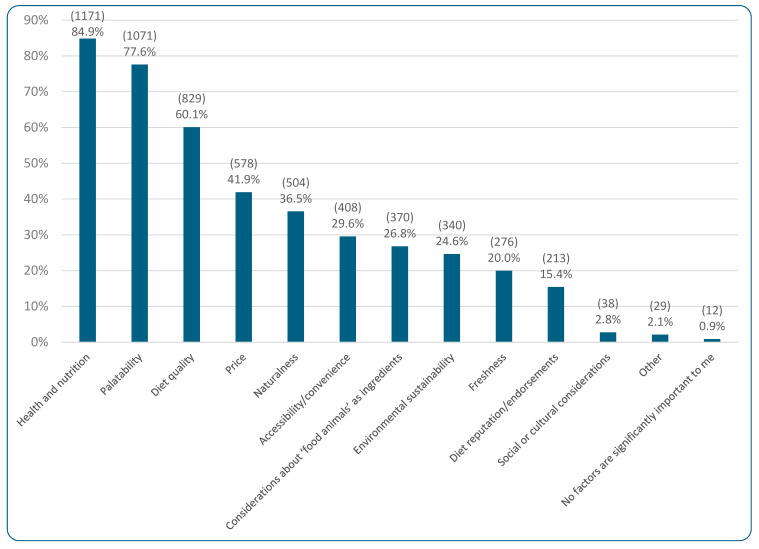
Distribution of current cat food purchasing determinants. Note: Multiple responses were possible; thus, the percentages and counts represent the proportion of all respondents (n = 1380) that selected each answer option. Elaborations on the “Other” responses included variations on the extant answer options, such as whether the guardian’s cat would eat the food, whether the food was vegan, support for independent pet shops, and veterinary opinion; variety and suitability for a cat with no teeth were also stated.

The vast majority of respondents (95.5%, 1318/1380) were the primary decision makers for cat food purchases, with 4.5% (62/1380) playing a lesser role. Figure 6 portrays the most commonly selected factors of importance when choosing current cat diets, with “health and nutrition” being the most popular option (selected by 84.9% of respondents, 1171/1380). When asked which health/nutritional factors were specifically important to these 84.9% of respondents, 87.9% (1028/1170) selected “maintenance of pet health,” 63.2% (740/1170) selected “nutritional soundness,” 53.4% (625/1170) “life stage suitability,” 3.1% (36/1170) “performance on diet,” and 1.5% “other” (18/1170) such as the ability to reduce hairballs, percentage of meat/vegan/grain, and allergen free. When asked if there were any particular nutrients these respondents wanted included within cat food, 68.6% (495/722) stated “no” and 31.4% (227/722) stated “yes.” From the 223 respondents providing qualitative answers to their “yes” response, taurine (an essential dietary amino acid for cats) was overwhelmingly the most common response. Other common responses included ‘whatever is required for optimal health,’ or to meet dietary guidelines provided by AAFCO/NRC/FEDIAF (respectively, the American Association of Feed Control Officials, the (US) National Research Council, and the European Pet Food Industry Federation), omega oils, arachidonic acid, and natural nonsynthetic sources.

Of the 26.8% (370/1380) of respondents selecting considerations about ‘food’ animals as an important factor in cat food purchases (Figure 6), 362 elaborated further by selecting the “welfare of ‘food’ animals” as a key consideration (selected by 79.8%, 289/362). “Rights of ‘food’ animals” was selected by 68.8% (249/362) and “other” by 5.5% (20/362). Of the 15.4% (213/1380) of respondents selecting “diet reputation/endorsements” as important, 210 answered the question about which endorsements they would like. “A good reputation without specific endorsement” was selected by 55.2% (116/210) respondents, “endorsement by veterinarians” was selected by almost as many–53.3% (112/210), “endorsement by other veterinary staff” by 17.1% (36/210), and “endorsement by others” by 16.7% (35/210). “Endorsement by others” was expanded upon by 31 respondents; common answers included friends/family, other customers, and other cat professionals.

Of the 2.8% (38/1380) selecting “social or cultural considerations” as an important factor in cat food purchases (Figure 6), 37 expanded on this. The most common concern was “country of origin of ingredients or finished product” (selected by 62.2%, 23/37), followed by “employment of farmers/workers” (selected by 37.8%, 14/37). “Cultural or religious considerations” was selected by 24.3% (9/37) and “other” by 16.2% (6/37).

**Figure 7:**
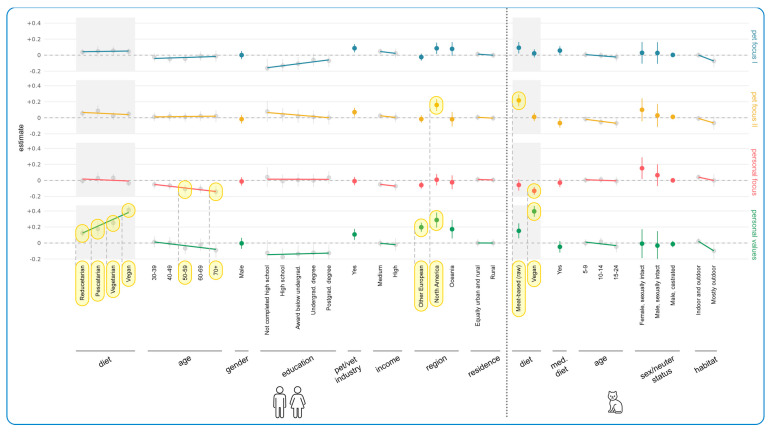
Linear regression results on the associations between human/cat demographic characteristics and current cat food purchasing determinants, relative to the reference characteristics for each group. Note: The reference characteristics for these humans and cats were, respectively: (human) omnivore, aged 18–29, female, doctoral degree, not in pet/vet industry, low income, UK- and urban-based; (cat) meat-based (conventional), no medical diet, aged 0–4, female, spayed, mostly indoors. The right *y*-axis purchasing determinant categories comprise the following subitems: Pet Focus I (health and nutrition, palatability, diet quality), Pet Focus II (naturalness, freshness, diet reputation), Personal Focus (price, convenience, social/cultural aspects), and Personal Values (concerns about ‘food’ animals or sustainability). Each dependent variable (e.g., Pet Focus I) comprises a score between 0 and 1, reflecting the share of its underlying set of items (see Figures S2 and S3) that respondents ticked in the questionnaire. Effects are depicted including 95% confidence intervals (not corrected for multiple testing). Effects that are significant after multiple testing correction are highlighted with yellow bubbles. Individual estimated effects of ordinal variables are grayed out in favor of an additional linear trend line. Effects of human and cat diet are highlighted through gray-shaded areas to reflect their separate estimation scheme (i.e., these effects were estimated while not controlling for further human demographic variables) as outlined in Section 2.2.

Figure 7 demonstrates the results of regression modeling, showing the associations between human/cat demographic characteristics and current cat food purchasing determinants. The most important results concerned human diet, human age, geographical region, and cat diet. Every human diet group (from reducetarian to vegan) rated Personal Values as significantly more important than the standard omnivore reference group, and in every case, these constituted effects. The more animal products were eliminated from guardians’ diets, the more importantly Personal Values were rated. This was most pronounced for vegan guardians who selected 41 percentage points more of the Personal Values items, compared to the reference group of omnivore guardians (+0.41, CI: [+0.37, +0.46], *p* < 0.0001). Figure S2 shows that these values applied to both sustainability concerns and concerns about the welfare of ‘food’ animals, across all of these categories.

Older guardians were less likely to be influenced by Personal Focus factors—and significantly so for two age groups. The share of Personal Focus items ticked by people aged 50–59 was on average 11 percentage points lower than the reference age group 18–29 (−0.11, CI: [−0.16, −0.05], *p* = 0.0269), while it was 14 percentage points lower for the 70+ age group (−0.14, CI: [−0.21, −0.07], *p* = 0.0128). In both cases these constituted effects. Figure S2 reveals this was due to price and convenience factors (not social factors).

Additionally, the share of Pet Focus II items selected by North Americans was on average 16 percentage points higher than those selected by UK guardians (+0.16, CI: [+0.09, +0.23], *p* = 0.0050), and the share of Personal Values items selected by North Americans (+0.29, CI: [+0.19, +0.38], *p* < 0.0001) and “Other Europeans” (from anywhere in Europe except the UK) (+0.20, CI: [+0.14, +0.25], *p* < 0.0001) was between 20 and 29 points higher than selected by UK respondents. In all cases these constituted effects. Figure S2 demonstrates that all options in each category scored quite highly.

Relative to guardians feeding conventional meat-based cat food, guardians feeding vegan diets were significantly less likely (−0.13, CI: [−0.18, −0.08], *p* < 0.0001) to find Personal Focus items important (especially price and convenience factors as shown in Figure S2), and significantly more likely (+0.40, CI: [+0.33, +0.46], *p* < 0.0001) to find Personal Values important. In both cases these constituted effects. Those feeding raw meat shared these tendencies, but they were less marked. In the latter case (Personal Values) only, this constituted a trend. Those feeding raw meat were, however, significantly more likely to find Pet Focus II important (+0.22, CI: [+0.14, +0.29], *p* < 0.0001) relative to those feeding conventional meat, and this constituted an effect. Figure S3 shows that this was due to concern for naturalness and freshness in particular.

### 3.3. Acceptance and Essential Characteristics of More Sustainable Cat Diets

#### 3.3.1. Acceptance of More Sustainable Cat Diets

Those feeding conventional or raw meat-based diets (collectively 88.8%, 1226/1380) were asked which other diet types they would realistically consider feeding their cat, assuming all desired attributes for these diets could be met. Table 2 summarizes their responses. All alternatives remained unacceptable to 49.3% (597/1211) of those feeding conventional or raw meat-based diets. Cultivated meat-based cat food was chosen by 33.1% (401/1211). Vegan, insect-based, and vegetarian options were selected to relatively comparable extents (18.2%, 221/1211; 14.8%, 179/1211; and 13.8%, 167/1211, respectively).

**Figure 8:**
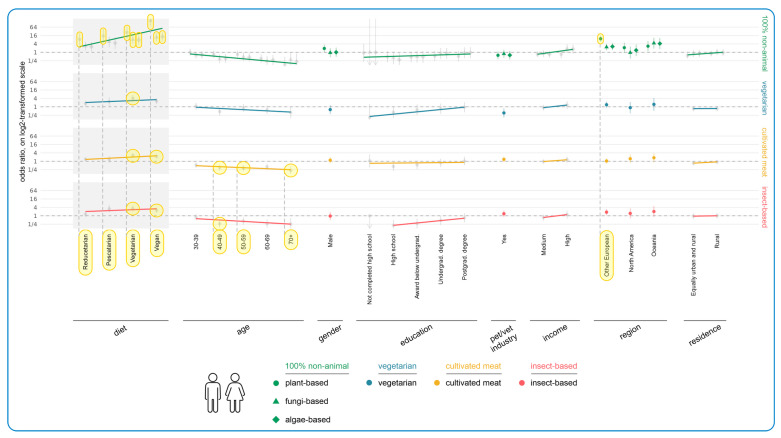
Logistic regression results on the associations between human demographic characteristics and the acceptance of more sustainable cat diets, among guardians currently feeding meat-based cat food (raw or conventional), relative to the reference characteristics for each group. Note: The reference characteristics for these people were: omnivore, aged 18–29, female, doctoral degree, not in pet/vet industry, low income, UK- and urban-based. Effects are depicted as odds ratios, including 95% confidence intervals (not corrected for multiple testing). Effects that are significant after multiple testing correction are highlighted with yellow bubbles. Individual estimated effects of ordinal variables are grayed out in favor of an additional linear trend line. Effects of human diet are highlighted through gray-shaded areas to reflect their separate estimation scheme (i.e., these effects were estimated while not controlling for further human demographic variables) as outlined in Section 2.2.

Figure 8 illustrates the results of regression modeling of the associations between human demographic characteristics and the acceptance of more sustainable cat diets, among guardians currently feeding meat-based cat food (raw or conventional). Most noteworthy were the tendencies associated with human diet and human age. There was an increasing acceptance of all 100% non-animal cat diets (especially plant-based) with decreasing human consumption of animal products. For instance, reducetarians had a 756% higher chance of accepting plant-based cat diets, compared to the omnivore reference category (OR = +756%, CI: [+222%, +2178%], *p* = 0.0052). This constituted an effect.

There was also a general slight upward trend of acceptance for other alternative diets, as guardians consumed fewer animal products. This was noteworthy for vegetarians, who were most accepting of vegetarian cat diets (OR = +300%, CI: [+139%, +559%], *p* < 0.0001), and for both vegetarians and vegans who were most accepting of cultivated meat-based cat food (vegetarian: OR = +157%, CI: [+72%, +283%], *p* = 0.0012; vegan: OR = +126%, CI: [+60%, +219%], *p* = 0.0010) and of insect-based cat food (vegetarian: OR = +235%, CI: [+103%, +451%], *p* = 0.0007; vegan: OR = +154%, CI: [+61%, +300%], *p* = 0.0192). In all cases these constituted effects.

As human age increased, there was a general decreasing level of acceptance of all 100% non-animal options, with four significant results. Considering cultivated meat-based cat food, age groups 40–49 (OR = −64%, CI: [−78%, −42%], *p* = 0.0101), 50–59 (OR = −68%, CI: [−80%, −47%], *p* = 0.0017), and 70+ (OR = −78%, CI: [−89%, −57%], *p* = 0.0022) were all significantly less accepting of this alternative relative to those in the reference category aged 18–29. Those aged 40–49 were also significantly less accepting of insect-based cat food (OR = −73%, CI: [−86%, −48%], *p* = 0.0244). In all cases these constituted effects.

**Figure 9:**
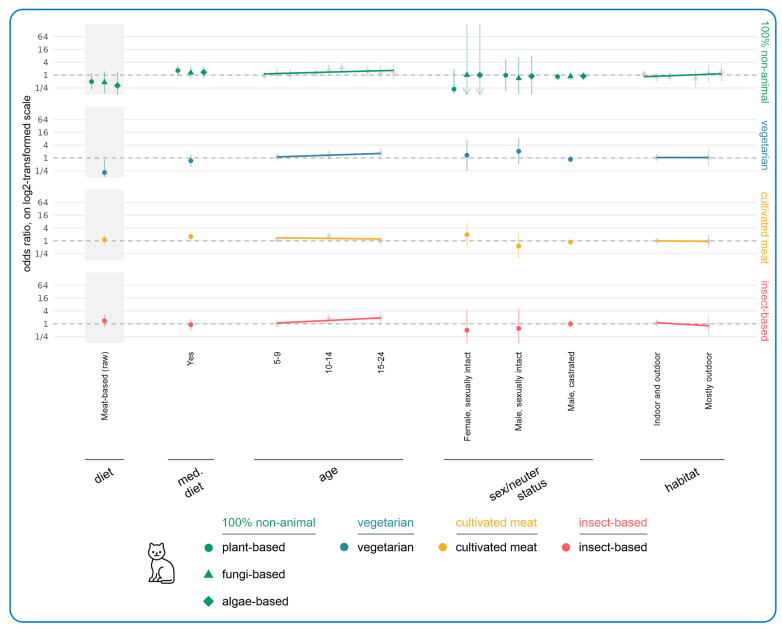
Logistic regression results on the associations between cat demographic characteristics and the acceptance of sustainable cat diets, relative to the reference characteristics for each group. Note: The reference characteristics for these cats were: meat-based (conventional), no medical diet, aged 0-4, female, spayed, mostly indoors. Effects are depicted as odds ratios, including 95% confidence intervals (not corrected for multiple testing). No effect was significant after correction for multiple testing. Individual estimated effects of ordinal variables are grayed out in favor of an additional linear trend line. Effects of cat diet are highlighted through gray-shaded areas to reflect their separate estimation scheme (i.e., these effects were estimated while not controlling for further human demographic variables) as outlined in Section 2.2.

Other Europeans had a significantly higher acceptance of plant-based cat diets than guardians in the UK (OR = +837%, CI: [+490%, +1387%], *p* < 0.0001). This constituted an effect. Similarly, there was also an upward tendency of acceptance of all more sustainable cat foods as education level increased, but this was not significant. For instance, guardians with only high school education were 52% less likely to accept plant-based cat food, compared to the reference category of guardians with a doctoral degree (OR = −52%, CI: [−81%, +18%], *p* > 0.9999). Additionally, there was a tendency towards lower acceptance of vegetarian cat foods among those working in the veterinary/pet sector (OR = −64%, CI: [−83%, −23%], *p* > 0.9999). This constituted a trend. When considering associations with cat demographic characteristics, there were no noteworthy associations (Figure 9) other than guardians feeding raw meat-based cat food being less likely to accept vegetarian cat food. This was not significant (OR = −79%, CI: [−95%, −14%], *p* > 0.9999) but did constitute a trend.

#### 3.3.2. Essential Characteristics of Alternative Cat Diets

**Figure 10:**
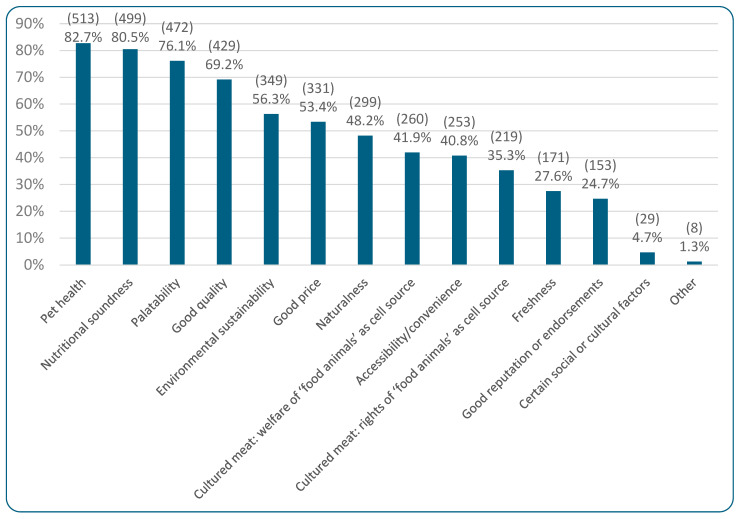
Distribution of attributes of sustainable cat food alternatives considered essential by guardians currently feeding conventional/raw meat-based cat food. Note: These attributes were considered essential in order for these respondents to realistically consider choosing alternative options. Multiple responses were possible; thus, the percentages and counts represent the proportion of all respondents (n = 620) selecting each answer option. Among these respondents are six respondents who deemed all options unacceptable, but who also simultaneously selected one of the alternatives as acceptable (cultivated meat, n = 4; vegan, n = 1; insect-based, n = 1). Elaborations on “Other” included veterinarian approval, cat choice/preference, suitability for sensitive cats, and nutritionally sound beyond minimum requirements.

Figure 10 illustrates the characteristics that more sustainable alternatives diets would need to supply, to be chosen by the 51.2% (620/1211) of guardians currently feeding meat-based cat food who would realistically consider purchasing the aforementioned alternatives. Among these respondents are six respondents who deemed all options unacceptable, but who also simultaneously selected one of the alternatives as acceptable (cultivated meat, n = 4; vegan, n = 1; insect-based, n = 1). “Confidence about pet health” (82.7%, 513/620) and “confidence about nutritional soundness” (80.5%, 499/620) were the top two attributes. When the respondents selecting the latter option were asked whether there were specific nutrients alternative foods would need to provide, 67.7% (338/499) said “no,” with the remainder stating “yes” (32.3%, 161/499). Those selecting “yes” were asked which nutrients would be necessary. Among 162 responses, common responses included “whatever is required for good health,” taurine, omega oils, FEDIAF-/NRC-/AAFCO-required nutrients, protein, and essential amino acids. Of the 24.7% (153/620) selecting “good reputation or endorsements” as essential, 66.0% (101/153) selected “endorsements by veterinarians,” 37.9% (58/153) selected “a good reputation without specific endorsements would be enough,” 19.6% (30/153) selected “endorsement by other veterinary staff,” and 14.4% (22/153) selected “endorsement by others.” Of the 22 selecting “endorsement by others,” when asked which others, common responses included customers, cat paraprofessionals, and friends/family. Of the 4.7% (29/620) selecting “certain social or cultural factors,” when asked what these were, 72.4% (21/29) selected “country of origin of ingredients or finished product” as an important factor, 62.1% (18/29) selected “employment of farmers and workers,” and 13.8% (4/29) selected both “cultural or religious factors” and “other” (e.g., veganism, free-range, and support for small pet shops).

**Figure 11:**
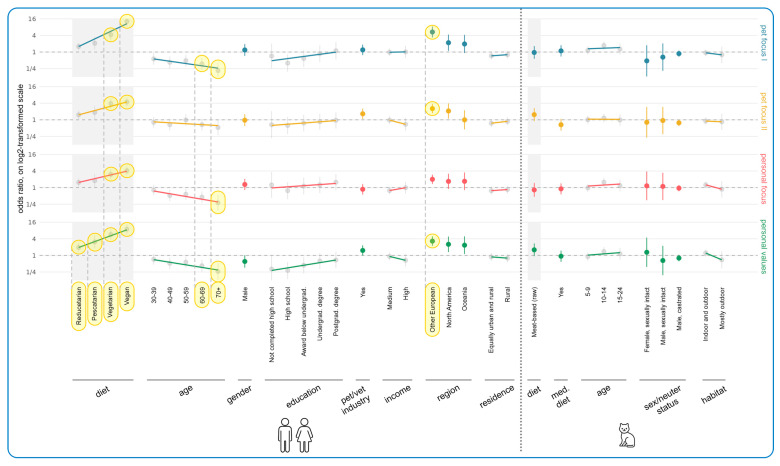
Logistic regression results on the associations between human/cat demographic characteristics and characteristics of more sustainable cat diets considered essential, among guardians currently feeding meat-based cat food (raw or conventional), relative to the reference characteristics for each group. Note: The reference characteristics for these humans and cats were, respectively: human: omnivore, aged 18–29, female, doctoral degree, not in pet/vet industry, low income, UK- and urban-based; cat: meat-based (conventional), no medical diet, aged 0-4, female, spayed, mostly indoors. The right *y*-axis essential characteristic categories comprise the following subitems: Pet Focus I (health, nutrition, palatability, diet quality), Pet Focus II (naturalness, freshness, diet reputation), Personal Focus (price, convenience, social/cultural aspects), and Personal Values (sustainability and animal welfare/rights regarding source animals used for cultivated meat-based cat food). Each dependent variable (e.g., Pet Focus I) reflects the information if at least one item among its underlying set of items (see Figures S4 and S5) was ticked in the questionnaire. Effects are depicted as odds ratios, including 95% confidence intervals (not corrected for multiple testing). Effects that are significant after multiple testing correction are highlighted with yellow bubbles. Individual estimated effects of ordinal variables are grayed out in favor of an additional linear trend line. Effects of human and cat diet are highlighted through gray-shaded areas to reflect their separate estimation scheme (i.e., these effects were estimated while not controlling for further human demographic variables) as outlined in Section 2.2.

Figure 11 illustrates the results of regression modeling of the associations between human and cat demographic characteristics, and characteristics of more sustainable cat diets considered essential, among guardians currently feeding meat-based cat food (raw or conventional). Human diet, human age, and region were again most strongly associated with these essential characteristics. There was a general increasing tendency of finding all categories important with decreasing animal product consumption, relative to omnivore guardians. This was significant for all diets for Personal Values: vegan (OR = +793% (CI: [+519%, +1187%], *p* < 0.0001), vegetarian (OR = 474%, CI: [+279%, +769%], *p* < 0.0001), pescatarian (OR = +207%, CI: [+78%, +429%], *p* = 0.0092), and reducetarian (OR = +94%, CI: [+36%, +176%], *p* = 0.0404). For vegetarians and vegans, differences were also significant for the other three categories. Vegetarians had a 326% higher chance of considering the Pet Focus I category important (OR = +326%, CI: [+185%, +537%], *p* < 0.0001), a 290% higher chance of considering the Pet Focus II category important (OR = +290%, CI: [+159%, +486%], *p* < 0.0001), and a 203% higher chance of considering the Personal Focus category important (OR = +203%, CI: [+102%, +353%], *p* < 0.0001), compared to omnivores. Similarly, vegan guardians had higher chances of considering the Pet Focus I (1196% (OR = +1196%, CI: [+756%, +1863%], *p* < 0.0001)), Pet Focus II (345% (OR = +345%, CI: [+213%, +531%], *p* < 0.0001)), and Personal Focus (311% (OR = +311%, CI: [+191%, +479%], *p* < 0.0001)) categories to be important, relative to omnivore guardians. In all of these cases, these tendencies constituted effects. Figure S4 demonstrates that all individual factors within each category were similarly important.

As guardian age increased, all categories became less important. Apart from Pet Focus II, this was significant for the 70+ age group with respect to Pet Focus I (OR = −79%, CI: [−89%, −60%], *p* = 0.0003), Personal Focus (OR = −72%, CI: [−85%, −46%], *p* = 0.0268), and Personal Values (OR = −74%, CI: [−87%, −48%], *p* = 0.0250). It was also significantly important for the 60–69 age group within the Pet Focus I category (OR = −63%, CI: [−78%, −39%], *p* = 0.0197). All of these tendencies constituted effects. Figure S4 demonstrates that the individual factors within each category all scored similarly, although palatability seemed to retain more value than other factors.

In contrast, all categories increased in importance with increasing education (though not significantly), with Figure S4 demonstrating that all individual items scored similarly. For instance, guardians with only high school education were 60% less likely to find the Pet Focus I category important, compared to people with a doctoral degree (OR = −60%, CI: [−81%, −17%], *p* > 0.9999). This constituted a trend. Additionally, Other European guardians were significantly more committed to Pet Focus I (OR = +433%, CI: [+236%, +744%], *p* < 0.0001), Pet Focus II (OR = +155%, CI: [+72%, +277%], *p* = 0.0005), and Personal Values (OR = +230%, CI: [+122%, +389%], *p* < 0.0001) compared to UK guardians. All of these constituted effects. Figure S4 shows this applied to all factors but especially to health, naturalness, and sustainability. There were no or minimal tendencies relating to the cat demographic variables.

### 3.4. Cat Diet Information Sources

**Figure 12:**
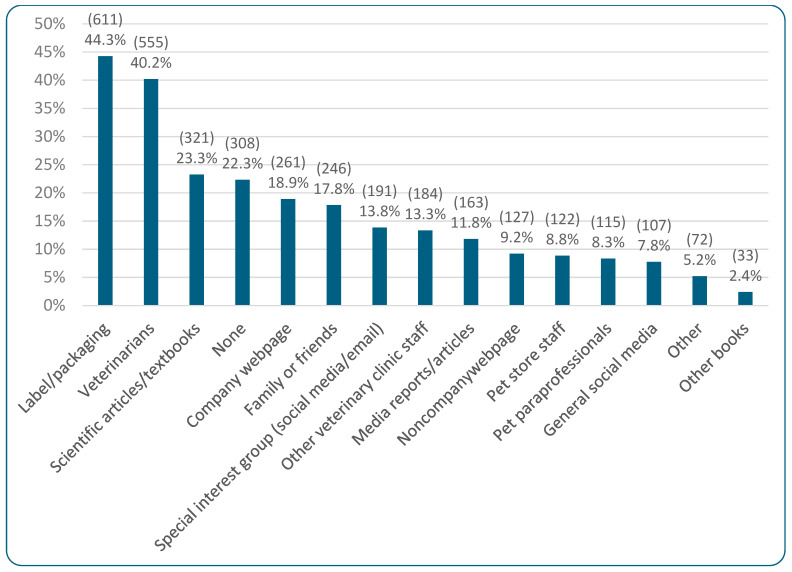
Distribution of information sources significantly influencing respondents’ cat diet choices. Note: Multiple responses were possible; thus, the percentages and counts represent the proportion of all respondents (n = 1380) selecting each answer option. “Other” responses included cat charity recommendations/practice, vegan charity recommendations, advertisements, research, behavioral feedback from cats, and experts in cat nutrition.

Figure 12 demonstrates the information sources that respondents selected as significantly influencing their cat food choices. “Label/packaging” was the most commonly selected option, being chosen by 44.3% (611/1380). Additionally, 80.8% (1115/1380) reported not having received any nutritional recommendations from veterinary clinic staff during the previous year, with 17.0% (234/1380) reporting that they had received such recommendations and 2.2% (31/1380) stating they were unsure. Of those receiving veterinary recommendations, 229 left comments regarding what particular advice they received. Responses are tabulated in Table 5. Compliance with the veterinary advice received was reported as “good” by 59.8% (137/229), “medium” by 20.5% (47/229), and “poor” by 19.7% (45/229).

**Figure 13:**
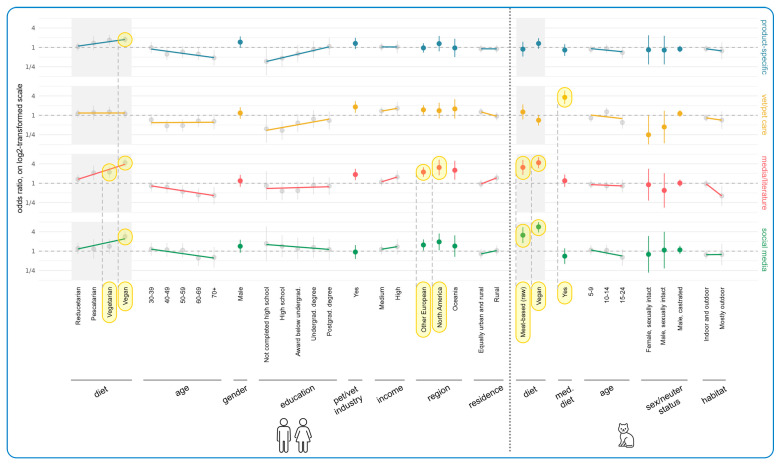
Logistic regression results on the associations between human/cat demographic characteristics and dietary information sources, relative to the reference characteristics for each group. Note: The reference characteristics for these humans and cats were, respectively: (human) omnivore, aged 18–29, female, doctoral degree, not in pet/vet industry, low income, UK- and urban-based; (cat) meat-based (conventional), no medical diet, aged 0–4, female, spayed, mostly indoors. The right y-axis information source categories comprise the following subitems: Product-Specific (label/packaging, company webpage), Vet/Pet Care (veterinarians, other vet clinic staff, pet store staff, pet paraprofessionals), Media/Literature (scientific literature, media reports, non-company webpages, other books), and Social Media (online special interest groups, general social media). Each dependent variable (e.g., Product-Specific) reflects the information if at least one item among its underlying set of items (see Figures S6 and S7) was ticked in the questionnaire. Effects are depicted as odds ratios, including 95% confidence intervals (not corrected for multiple testing). Effects that are significant after multiple testing correction are highlighted with yellow bubbles. Individual estimated effects of ordinal variables are grayed out in favor of an additional linear trend line. Effects of human and cat diet are highlighted through gray-shaded areas to reflect their separate estimation scheme (i.e., these effects were estimated while not controlling for further human demographic variables) as outlined in Section 2.2.

Figure 13 demonstrates the results of regression modeling of the associations between human/cat demographic characteristics and information sources used by guardians when making decisions about cat diets. Human diet was associated with the most significant effects. There was a general increasing tendency toward using all information source categories (barring Vet/Pet Care) as more animal products were eliminated from guardians’ diets. This was significant for vegans: Product-Specific (OR = +76%, CI: [+33%, +134%], *p* = 0.0156), Media/Literature (OR = +333%, CI: [+219%, +488%], *p* < 0.0001), and Social Media (OR = +183%, CI: [+98%, +303%], *p* < 0.0001). It was also significant for vegetarians in regard to Media/Literature (OR = +121%, CI: [+47%, +231%], *p* = 0.0235). In all cases these constituted effects. Figure S6 shows similar scorings for all individual factors included within these categories, although there was a particularly high score for ‘other books’ (in the Media/Literature category) from vegans. Other European (OR = +122%, CI: [+58%, +214%], *p* = 0.0010) and North American (OR = +208%, CI: [+77%, +436%], *p* = 0.0129) guardians were also significantly more likely to use Media/Literature. In both cases these constituted effects. Figure S6 shows that ‘other books’ were particularly favored.

There were also tendencies regarding age group and educational level, although results were not significant. Use of all information source categories seemed to decline with age; for instance, guardians aged 70+ were 53% less likely to use Product-Specific sources (OR = −53%, CI: [−72%, −18%], *p* > 0.9999) and 59% less likely to use Media/Literature (OR = −59%, CI: [−78%, −24%], *p* = 0.8055) relative to the 18–29 age group. In both cases these constituted trends. Figure S6 shows similar scorings for all individual factors included within these categories. In contrast, the use of some information sources increased with more education. For instance, guardians with only high school education were 54% less likely to use Product-Specific sources (OR = −54%, CI: [−75%, −13%], *p* > 0.9999) and 66% less likely to use Vet/Pet Care sources (OR = −66%, CI: [−82%, −34%], *p* = 0.2390), than guardians with a doctorate. In both cases these constituted trends. Figure S6 shows that these trends were particularly pronounced in regard to “label/packaging” (Product-Specific) and “pet store staff” and “pet paraprofessionals” (Vet/Pet Care). Additionally, guardians working in the pet/vet industry were over 80% more likely to use Vet/Pet Care sources (OR = +82%, CI: [+20%, +176%], *p* = 0.8119) and Media/Literature (OR = +86%, CI: [+24%, +179%], *p* = 0.4981), compared to people not working in this industry. Both of these also constituted trends.

In terms of cat demographic characteristics, Figure 13 also demonstrates that Media/Literature was selected significantly more by guardians feeding raw meat (OR = +210%, CI: [+81%, +431%], *p* = 0.0073) and vegan diets (OR = +333%, CI: [+190%, +546%], *p* < 0.0001), as was Social Media (raw meat: OR = +212%, CI: [+78%, +449%], *p* = 0.0138; vegan: OR = +466%, CI: [+280%, +742%], *p* < 0.0001), relative to guardians feeding a conventional diet. These all constituted effects. Figure S7 shows “other books” were particularly favored within the Media/Literature category and “special interest groups” were particularly favored within the Social Media category. It also shows the significant result of using Vet/Pet Care for cats progressing onto a medical diet (OR = +261%, CI: [+113%, +484%], *p* < 0.0001). This constituted an effect. Figure S7 shows that “advice from vets” was particularly important. There were also tendencies for sexually intact cats (both males and females) to use Vet/Pet Care less compared to female spayed cats (male, sexually intact: OR = −57% (CI: [−86%, +38%], *p* > 0.9999); female, sexually intact: OR = −75% (CI: [−94%, +0.004%], *p* > 0.9999)).

## 4. Discussion

This section discusses the most important results regarding each of the three parts of this research. Current feeding practices and purchasing determinants are first discussed, followed by acceptance of alternative sustainable cat food options, and finally, cat food information sources used by guardians. The section finishes with consideration of this study’s limitations, and with recommendations for the pet food industry and veterinary staff.

### 4.1. Current Feeding Practices and Purchasing Determinants

Nearly 90% (89.4%, 1234/1380) of cat guardians in this study used commercial feed for the bulk of their cats’ diets, with almost 73% (1003/1380) purchasing cat food from a store or online (not direct from the manufacturer). There was a three-way and roughly even split between use of wet food, dry food (kibble), or equal proportions of wet/dry. Conventional meat-based feed was used by over 84% of cat guardians (1162/1380). These statistics are similar to those found in other studies (e.g., Schleicher et al. [35]; Dodd et al. [36]), though kibble (dry food) was the predominant form of commercial feed provided to cats in these other studies. Differences in results could be explained by the fact that Schleicher et al. [35] combined dog and cat data together. Different question formats could also cause differences or ambiguities in the results; for instance, cat owners in Dodd et al.’s [36] study were asked to indicate the frequency of use for each different food type rather than to simply select the main type used, and they also had a far greater number of North American respondents than British. There was also a far higher proportion of cats already being fed a vegan diet in the present study (over 9%, 126/1380) relative to the 0.7% (11/1542) studied by Dodd et al. [36], due to the purposive sampling used in the present study.

Similar to Dodd et al. (personal correspondence) [36] who found 5% of cat guardians were feeding homemade food daily, of note was that over 4% (4.1%, 57/1380) of cat guardians from the current study stated that their cats’ diets comprised over 50% homemade food, with over 70% (70.2%, 40/57) of these guardians stating they did not use a recipe. This strongly suggests a welfare risk for these cats regarding whether they received nutritionally sound diets; this concern remains even if a recipe was used and regardless of whether the homemade food was meat-based or not [52].

This concern remains despite some discrepancies in the reporting of homemade cat food as the predominant cat food choice. Respondents had multiple opportunities to indicate the extent of their use of homemade cat food, and their answers did not always align. For instance, respondents were also asked about the source of the majority of their cat food, but “Diet is 50% or more homemade” was only selected by 1.6% (22/1380). Additionally, in Table 3, the combined answers for 0–49% of cat food being sourced commercially (i.e., indicating over 50% of cat food was homemade) amount to 8.8% (122/1380). The aforementioned 4.1% figure appears to be the most reliable among these three figures; however, because (1) it was arrived at by a dedicated stand-alone question about homemade diets, and (2) it echoes figures from extant literature as mentioned above. Nevertheless, future research should confirm this.

The top five considerations when purchasing current cat diets were cat health/nutrition (selected by 84.9%, 1171/1380), palatability (77.6%, 1071/1380), diet quality (60.1%, 829/1380), price (41.9%, 578/1380), and naturalness (36.5%, 504/1380). This is broadly consistent with the top three factors of nutritional soundness, palatability, and quality found elsewhere in the literature (e.g., in O’Halloran et al. [38]; Schleicher et al. [35]). However, there is disagreement over the fourth and fifth most important purchasing determinants relating to cat food; the present study affirmed price to be within the top five (akin to Naughton et al. [37]; Dodd et al. [16]), but *not* ingredients used (akin to Naughton et al. [37], but dissimilar to Schleicher et al. [35]). The present study also established naturalness as one of the most important factors; this concurred with the results of Dodd et al. [16]. Similarly, Dodd et al. [16] found *un*naturalness to be the top factor predicting against feeding vegan diets to companion animals, at least among vegan guardians.

Most striking was how strongly associated guardian diet was to cat diet; almost 97% of guardians feeding their cats a vegan diet (96.8%, 122/126), were vegan themselves—only four nonvegans fed their cats a vegan diet (two vegetarians, one standard omnivore, and one reducetarian). This is comparable to other studies (e.g., Dodd et al. [16]). Additionally, almost 35% (122/361) of vegans *currently* fed their cats a vegan diet. This relates to the strong association found between Personal Values (i.e., concerns for food animals and sustainability as purchasing determinants) and human diet group; every human diet group (from reducetarian to vegan) rated Personal Values as significantly more important than the standard omnivore reference category. Moreover, the more animal products were eliminated from the diet, the more importantly Personal Values were rated. This suggests that Personal Values need to be a high priority for cat guardians before they will commit to feeding nutritionally sound vegan diets to their cats. This pattern was also reflected in the rating of Personal Values by guardians of vegan-fed cats as significantly more important relative to guardians of conventional meat-based cats; such guardians of vegan-fed cats also found Personal Focus considerations significantly less important, relative to guardians of conventional meat-based cats.

Male guardians comprised 8.8% (121/1380) of the respondents. They had a 112% higher chance of feeding their cats vegan diets compared to female cat guardians. This was unexpected as numerous studies have demonstrated females to be more interested in animal welfare, veganism, and environmental protection [53]. However, some studies have shown that males are, generally speaking, more assertive and more likely to seek excitement [54]. Thus, as feeding cats nutritionally sound vegan diets still does not enjoy broad societal approval (e.g., by 2024, the British Veterinary Association had affirmed the suitability of nutritionally sound vegan diets for dogs, but not for cats [55]), it may be that male cat guardians are more willing to feed their cats nutritionally sound vegan diets currently. Whilst concerning a different animal species, male guardians of chickens, although again being underrepresented among the survey respondents, have also been found to be more regular users of hormonal implants in companion hens to decrease health problems associated with egg laying, which is a similarly fringe healthcare approach [56].

The region cat guardians live in also seems to have had an impact on feeding cats vegan diets, as well as on purchasing determinants. Compared to UK residents, North Americans had a 405% higher chance of feeding their cats a vegan diet, and North Americans found both Pet Focus II (i.e., diet naturalness, freshness and reputation) and Personal Values significantly more important as cat food purchasing determinants, relative to UK guardians. These two factors may be related; alternatively, the higher proportions of North Americans feeding a vegan diet may relate to the larger size of the vegan pet food industry in the US, relative to the UK [57]. Conversely, Dodd et al. [16] found that respondents in the UK had more concerns about feeding meat-based diets than guardians in the US. More research could try to further explore these apparently conflicting results. And although cost was important to many guardians—being ranked fourth as a purchasing determent—higher incomes did not seem to result in other purchasing determinants becoming more important.

While age initially increased the likelihood of feeding cats vegan diets, up to and including the 40–49 age group, there was a tendency of older cat guardians having a reduced likelihood of feeding their cats a vegan diet. (As noted, almost all guardians feeding vegan diets, and all guardians included within this analysis, were themselves vegan). This is aligned with studies demonstrating more openness among younger age groups [58]. However, the 50–59 and 70+ age groups (for all human dietary categories) were significantly less likely to be influenced by Personal Focus (i.e., price, convenience or social/cultural) considerations, relative to the 18–29 reference age group. Thus, there may still be potential in some older age groups for considering nutritionally sound vegan diets for cats, as at least cost and convenience are less likely to be barriers. This could be due to some older people (at least the 70+ age group) being more likely to have more spare time and money [59]. Unsurprisingly, there was a declining trend to feed cats vegan diets among guardians of older cats, likely due to long entrenched habits and increased health concerns.

### 4.2. Acceptance of More Sustainable Cat Diets

Approximately 50% (51.2%, 620/1211) of guardians currently feeding meat-based cat food (conventional or raw) found at least one of the alternative sustainable cat food options listed to be acceptable. Cultivated meat-based cat food was the most popular, selected by over 33% (401/1211). Vegan cat food was the next most common choice, although it was selected to a similar degree to insect-based and vegetarian cat food (all within 14–18%). However, because some of those selecting algae-based (9.7%, 117/1211) and fungi-based cat food (10.4%, 126/1211) may not have additionally selected vegan cat food (18.2%, 221/1211) (perhaps not realizing this term also applied), the true proportion of respondents considering vegan cat food acceptable may actually be higher, when considering that vegan diets actually include algae- and fungi-based diets—indeed all diets that exclude animal products.

Other studies have found greater willingness to feed vegan diets among all guardians not yet doing so, than this study has; for instance, it stands at 35% (1083/3130) for all guardians not currently feeding vegan (plant-based) in the study by Dodd et al. [16], and 32% (~160/500) in a survey by The Vegan Society [60]. Reasons for the lower rate within the current study could be first that our study focused exclusively on cats, versus both dogs and cats in the studies cited, and vegan diets are generally more accepted for dogs. Second, the present study enquired about the acceptance of numerous alternative more sustainable cat food options—not exclusively vegan diets. The presence of more answer options may have reduced the number of guardians who selected “vegan.” Third, conceivably, “vegan” cat food terminology used in the present study may potentially repel nonvegan guardians, due to the politicized nature of the term “vegan” [61]. Nevertheless, it is very encouraging that this study found that over 50% (620/1211) of guardians were open to alternative more sustainable cat food options as a whole.

When guardians feeding conventional or raw meat-based diets (who found at least one of the sustainable cat food options acceptable) were asked about the characteristics these more sustainable diets needed to offer in order to be chosen, the top answers were quite comparable to the most important purchasing determinants of *current* cat food choices, i.e., health/nutrition, palatability, and quality. However, the environmental sustainability of cat foods rose to become the fifth most important characteristic, ahead of both cost and naturalness. It is clear that when marketing more sustainable cat foods, environmental sustainability should be considered an important product attribute, and whilst cost remains important, its importance becomes relatively reduced. This potentially confirms Dodd et al.’s [16] finding of cost actually increasing as a concern with higher likelihood of feeding vegan diets; it is possible that cost diminishes as a concern among those who do not feed vegan diets to their cats. Similarly, the (un)naturalness of vegan cat diets may actually be more of a leading concern for vegan than nonvegan guardians.

The more that animal produce was removed from human diets, the greater the acceptance of alternative more sustainable cat food options (especially 100% non-animal options) among those currently feeding conventional or raw meat-based cat food. Similarly, the more animal produce was removed from human diets, the more important all categories of essential attributes of alternative cat foods became, especially Personal Values. Strong associations between guardian diet and acceptance of alternative diets have also been found in other studies (e.g., Dodd et al. [16]). It may be the case that there is less cognitive dissonance among those who eschew animal produce to lesser or greater degrees, which may enable them to more readily entertain the prospect of switching. Indeed, this prospect is further supported by the success of some authors (e.g., Oven et al. [62]) in mapping the justifications from animal guardians for continuing with feeding companion animals conventional or raw meat-based diets, onto the 4Ns—the notion that feeding meat to companion animals is considered to be normal, natural, necessary, and nice. These 4Ns originally summarized justifications from omnivorous humans for their continued consumption of animals [63].

Predictably, an increase in guardian education levels also led to more support for all more sustainable cat food options, and to the increasing importance of all categories of alternative cat food attributes considered essential, for these diets to be chosen. Despite some suggestion of the education–environmental concern connection dissolving [64], it may still apply in relation to nutritionally sound vegan cat diets due to the relative novelty of this topic. Additionally, the positive association between education levels and veganism still remains strong [65].

Other Europeans were shown to have a significantly higher acceptance of vegan cat diets relative to guardians in the UK. They also found Pet Focus I (i.e., health and nutrition, palatability, and diet quality), Pet Focus II (i.e., naturalness, freshness, diet reputation), and Personal Values significantly more important as essential characteristics of alternative cat foods. The reasons for these results are unclear.

As human age increases, there was an (often-significant) tendency of less acceptance of alternative cat food options among those currently feeding meat-based cat food (conventional or raw), and all categories of essential attributes of alternative cat foods were considered less important. This concurs with a tendency toward more openness to new experiences among younger age groups [58]. However, this contradicts the finding by Dodd et al. [16] that concerns about feeding vegan (plant-based) diets to companion animals reduced with increasing human age. Terminological or question-phrasing differences and not distinguishing between dog/cat data within the Dodd et al. [16] study may be causes. Additionally, there was a trend of lower acceptance of vegetarian (as distinct from vegan) cat foods among those working in the veterinary/pet sector. This may reflect recent research and communication demonstrating health and environmental benefits of nutritionally sound *vegan*, rather than *vegetarian*, cat foods (e.g., Knight [66]; Knight et al. [29]). Thus, there may be less certainty about the efficacy of vegetarian cat foods or their impacts (e.g., on sustainability outcomes).

### 4.3. Cat Diet Information Sources

Labels/packaging of cat food (selected by 44.3%, 611/1380) and veterinarians (selected by 40.2%, 555/1380) were—by some margin—the two most commonly used sources of information when making purchasing decisions about cat food. In contrast, the third most popular information source (scientific articles/textbooks) was only selected by just over 23.3% (321/1380). Given that veterinarians are still prioritized as a top information source, it is vital they are provided with current scientific information regarding sustainable cat diets, and especially nutritionally sound vegan diets, as these are the most widely available.

For statistical modeling purposes, the individual information source options were grouped into the categories: Product-Specific (label/packaging, company webpage), Vet/Pet Care (veterinarians, other vet clinic staff, pet store staff, pet paraprofessionals), Media/Literature (scientific literature, media reports, non-company webpage, other books), and Social Media (special interest group online, general social media). Interestingly, the more animal produce was removed from human diets, the more highly each category was rated—apart from Vet/Pet Care. This tendency was significant for vegans (and vegetarians for Media/Literature). Relatedly, guardians feeding *un*conventional cat diets (vegan or raw meat) were found to use Media/Literature and Social Media significantly more than guardians feeding conventional diets, with “special interest groups” on social media being particularly used. These two points could suggest at least a partial lack of trust in, or at least a lack of enthusiasm for, veterinary professionals by guardians who choose less conventional cat diets. This lack of trust in veterinary staff by guardians choosing less conventional options has been demonstrated and discussed elsewhere (e.g., Schleicher et al. [35]).

### 4.4. Limitations

Due to the overrepresentation of females, British residents, and vegan guardians, caution should be taken before extrapolating the reported population-level relative frequencies of specific subgroups from this study to the general population of any particular country. Caution should also be taken when interpreting results from North American cat guardians as that sample size was quite small. There was also a great overrepresentation of vegans, compared to the general population. While this was intentional so as to secure sufficient, meaningful data from this group of cat guardians, it may mean the reported subgroup frequencies are less transferable to general populations. All associations reported in this work were estimated using regression models. As regression is a conditional analysis, the overrepresentation of the aforementioned sub-groups was not expected to substantially bias these findings. The results may be less transferable to niche cat guardians such as of cats with medical needs or cats used for breeding. Dedicated research should be conducted for specialist subpopulations. Similarly, it should be noted that guardians of cats fed on medical diets were asked to answer questions based on their cats during the year prior to commencing their medical diet. This should be considered when interpreting results from this group. Additionally, the term “nutritionally sound vegan diets” was not applied in the questionnaire, with only “vegan diet” being used. It is conceivable that terminology such as “nutritionally sound vegan diets,” which is becoming more common (e.g., Knight et al. [29]; Nicholles & Knight [67]), might help to reassure some cat guardians, resulting in an even higher level of acceptance of such vegan diets than indicated within this study. Similarly, perceptions and product availability have evolved since this 2020 survey was conducted, particularly regarding cultivated meat, with greater awareness of these diets and their potential benefits. Finally, a convenience sample with reliance on self-reported data from English-speaking guardians is always susceptible to biases [68]. Such risks were minimized by gathering a very large sample size, and through use of purposive sampling to ensure a diverse range of views were captured. Lastly, the goodness-of-fit of our regression models (i.e., the AUC and R^2^ values) was (very) low in some cases. However, as outlined in our Statistical Analysis section, we do not believe that this hindered the interpretation of the estimated effect patterns and significances given our social science research framework.

### 4.5. Recommendations

The health of cats and the nutritional soundness of their diets were the leading purchasing determinants of diets currently fed. They were also the leading characteristics more sustainable cat foods would need to offer, in order for guardians to realistically consider choosing them. Additionally, veterinary staff and pet paraprofessionals were a key source of information about cat diets. For these two reasons, it is of paramount importance to continue gathering key institutional seals of approval for nutritionally sound vegan cat diets, and other sustainable alternatives, if seeking to optimize their uptake by cat guardians. Moreover, a potential lack of trust among vegans/vegetarians of diet advice from veterinarians highlights the need for the veterinary community to foster improved client–veterinarian relations (and thus animal welfare), by ensuring staff remain abreast of up-to-date information, and by working *with* clients seeking to use alternative diets, rather than against them, where possible while still upholding animal welfare. The present study demonstrated that nearly 50% (49.3%, 597/1211) of cat guardians currently feeding meat-based cat food (conventional or raw) found all more sustainable cat diet alternatives to be unacceptable. This is a high proportion, and more educational outreach is recommended to address this.

More research is also recommended into optimal naming of vegan cat food. If vegan guardians are the primary target group, “nutritionally sound vegan cat food” may prove most resonant; however, there may be greater acceptance of vegan cat food among nonvegan guardians if vegan cat food is described as “plant-based.” This would echo trends in human food purchasing behavior. “Reducetarian” messaging could also be applied to cat-feeding habits.

Cultivated meat-based cat food was the most popular sustainable cat food option; thus, efforts should continue to increase the availability of commercially viable products. Additionally, with nearly three quarters (74.9%, 1034/1380) of cat guardians feeding their cats treats outside of main meals, it is also recommended that industry prioritize innovation of vegan cat treats in addition to nutritionally sound vegan main meals. Similarly, other niche areas could be developed, such as nutritionally sound vegan food for senior cats, or other specialized diets.

Within further research, investigators are encouraged to consider terminology and survey designs consistent with those used within extant literature, to facilitate data comparisons. A related issue is that the list of options provided, or guardian motivations explored, is very long in some studies (e.g., Schleicher et al. [35]), but very short in others (e.g., Naughton et al. [37]).

## 5. Conclusions

By 2023 the global cat food industry had been valued at USD 31 billion. It was also forecast to grow by 4.4% annually until 2030. Among this projected growth is an array of current or developing alternatives to standard commercial meat-based cat food, such as diets based on raw meat, vegan ingredients, insects, proteins from fermented microorganisms, and cultivated meat. Potential benefits of some alternative cat foods include improved health outcomes, superior environmental sustainability, and reduced animal welfare and ethical concerns surrounding the use of ‘food’ animals.

This study of 1380 cat guardians analyzed cat food purchasing determinants, feeding patterns, and cat food information sources used by guardians. Crucially, it also investigated the acceptance of alternative more sustainable cat foods among cat guardians currently feeding meat-based diets (conventional or raw). Over 50% (620/1211) of cat guardians currently feeding conventional/raw meat-based cat food found at least one more sustainable alternative to be acceptable, with cultivated meat-based cat food being the most popular alternative, followed by nutritionally sound vegan diets. However, acceptance of alternatives was contingent upon these providing certain essential characteristics. The top five were (in order): good health outcomes, nutritional soundness, palatability, quality, and environmental sustainability. These essential characteristics largely mirrored current purchasing determinants of cat guardians, although environmental sustainability became more important, and cost and naturalness became marginally less important. It is important to emphasize that nearly 50% of cat guardians currently feeding conventional/raw meat-based cat food found none of the listed more sustainable alternative cat foods acceptable; hence targeted educational outreach to this group could be of benefit.

Almost all (bar four individuals) guardians feeding a vegan diet to their cats were vegan themselves, with male vegan guardians being 112% more likely than females to feed vegan diets. The most important information sources used by all guardians for making decisions about cat food were, by some margin, labels/packaging and veterinarians. Human diet was strongly associated with cat diet choices and attitudes; the more animal products were eliminated from cat guardian diets, the more Personal Values (e.g., ‘food’ animal and sustainability concerns) were prioritized in cat food purchases, and the less Personal Focus aspects (e.g., price and convenience) were. The more animals were removed from the human diet, the more highly each information source was also rated, and significantly so for vegans—except for Vet/Pet Care, which may indicate potential trust problems in respect to cat diets, between the veterinary profession and vegans.

## Figures and Tables

**Table 1:** Distribution of cat guardian demographic characteristics (n = 1380 in each category).

	Percentage	Frequency
**CONTINENTAL REGION**		
UK	70.5%	973
Other European	19.6%	270
North America	4.9%	67
Australia/New Zealand/Oceania	3.0%	42
Asia	1.2%	17
Other	0.7%	10
South America	0.1%	1
Africa	0.0%	0
		
**HIGHEST EDUCATIONAL LEVEL**		
Did not complete high school	2.3%	32
High school or equivalent	22.1%	305
College or University award < undergrad	24.5%	338
University undergraduate degree	26.8%	370
Postgrad < doctorate	20.3%	280
Doctoral degree	4.0%	55
		
**AGE CATEGORY**		
18–19	0.7%	9
20–29	13.4%	185
30–39	19.2%	265
40–49	17.2%	238
50–59	20.6%	284
60–69	20.1%	278
70+	8.8%	121
		
**HUMAN DIET**		
Omnivore	35.1%	484
Reducetarian	22.4%	309
Pescatarian	5.2%	72
Vegetarian	10.4%	144
Vegan	26.2%	361
Other	0.7%	10

**Table 2:** Distribution of current cat diets (n = 1380) and acceptance of more sustainable alternative diets among those (n = 1211) currently feeding conventional/raw meat-based cat food. Note: While fungi- and algae-based cat foods are vegan, the options listed reflect those offered to respondents within this survey. Respondents could select more than one option when answering this question concerning alternative options.

	Percentage	Frequency
**CURRENT CAT DIET**		
Meat-based—conventional	84.2%	1162
Vegan	9.1%	126
Meat-based—raw	4.6%	64
Meat-based—cultivated	0.7%	9
Unsure	0.6%	8
Mixture	0.5%	7
Vegetarian	0.2%	3
Insect-based	0.1%	1
		
**ACCEPTANCE OF MORE SUSTAINABLE CAT DIET**		
None of the options	49.3%	597
Meat-based—cultivated	33.1%	401
Vegan	18.2%	221
Insect-based	14.8%	179
Vegetarian	13.8%	167
Fungi-based	10.4%	126
Algae-based	9.7%	117

**Table 3:** Distribution of source of cat food purchases, type of cat food used, and percentage of commercial cat food used (n = 1380 in each category).

	Percentage	Frequency
**SOURCE OF MAJORITY PET FOOD**		
Other store (e.g., supermarket, grocery store, farmer’s market, pharmacy)	46.2%	637
Ordered online not directly from the manufacturer	26.5%	366
Pet store	16.7%	231
Direct from the manufacturer	4.1%	56
Veterinary clinic	3.6%	50
Diet is 50% or more homemade	1.6%	22
Other	1.3%	18
		
**PET FOOD CONSISTENCY**		
Commercial canned/pouch/moist/semi-moist	32.6%	450
An equal mix of dry food, with moist or raw	29.9%	413
Commercial dry kibble (i.e., does not require additional ingredients)	27.3%	377
Commercial dry premix (i.e., for use with additional ingredients)	3.8%	52
Commercial raw	2.0%	28
Other	1.8%	25
Home-prepared raw	1.2%	17
Home-prepared cooked	0.9%	12
Human food (home-prepared or commercial)	0.4%	6
		
**% COMMERCIAL**		
100%	42.8%	590
75–99%	36.8%	508
50–74%	11.6%	160
25–49%	4.2%	58
5–24%	1.5%	21
0–4%	3.1%	43

**Table 4:** Distribution of respondents’ use of cat treats (n = 1034 in each category). Note: Multiple responses were possible when selecting ‘type of treat.’ Thus, the percentages and counts represent the proportion of all respondents selecting each answer option. ‘Other’ answers (type of treat) commonly comprised wet cat food and specific examples of human food and/or commercial cat treats, such as cooked chicken and anti-hairball treats.

	Percentage	Frequency
**FREQUENCY OF SNACKS/TREATS**		
More than once a day	16.5%	171
Once a day	38.1%	394
More than once a week but less than once a day	29.7%	307
Once a week	9.0%	93
Less than once a week	6.7%	69
		
**TYPE OF TREAT**		
Other commercial treats	68.5%	708
Human food prepared at home	30.7%	317
Dental/oral bars or chewable sticks	30.1%	311
Human food from other sources	11.1%	115
Raw meat or bones	7.7%	80
Vegetables or fruit	7.2%	74
Other	7.1%	73

**Table 5:** Distribution of veterinary nutritional advice received (n = 229). Note: Respondents were able to state multiple recommendations.

	Frequency
**ADVICE**	
Sensitivity/prescription/condition diet	81
Weight reduction	39
Particular brands (e.g., Royal Canin, Hill’s Science Plan, Purina)	22
Hypoallergenic/elimination diets	18
Against feeding vegan	12
Use supplements/ensure sufficient protein, taurine, calcium, phosphorus, vitamin C, cranberries, L-Methionine, Pronefra drops, fiber	11
Only wet food	10
Only dry food	9
Grain-free	9
Advice on quantities/regularity	8
Premium cat foods	8
OK with feeding vegan	5
Mixed dry/wet	5
Raw meat	4
General advice	3
Sugar-free	3
Increase weight	2
Recommendation of brands stocked by vets	2
Vary food to avoid intolerances emerging	2
Low sodium	1
Not pet milk	1
Include grains	1
Do research	1
Hydrolyzed protein	1

## Data Availability

This study’s data, questionnaire, and the R code used for the data’s statistical analysis, are available at https://osf.io/nbepu (accessed 3 october 2025).

## Supplementary Materials

The following supporting information can be downloaded at https://www.mdpi.com/article/10.3390/ani15202984/s1, Figures S1–S7, and Tables S1–S3.
