# Supplementary material for: Consumer Acceptance of Sustainable Cat Diets: A Survey of 1380 Cat Guardians
Source: Animals (Basel). 2025 Oct 15;15(20):2984. doi: 10.3390/ani15202984 (PMC12560941; doi:10.3390/ani15202984)
Supplement: Supplementary file 1 [file animals-15-02984-s001.zip › animals-3806110-Supplementary Tables.pdf]

Supplementary Tables S1a-b, S2a-b, S3

Complementing the results section of the main paper, the following five tables summarize the key statistics from Figures 5, 7, 9–11, 13, and S1 regarding correlations between human and cat demographic variables, and key variables of the Research Questions (RQs). There is a table for each RQ. In each table, human demographics are covered first, followed by cat demographics. Additionally, the most noteworthy variables are listed first, though the order of the variable items follows the order of their display in the figures.

Table S1a. Key statistics relating to RQ1a (current feeding patterns).

Note: The data specifically concern Figure 5 regarding likelihood of vegan guardians to feed vegan cat food and Figure S1 regarding likelihood of guardians feeding meat, to feed raw meat-based cat food. Effects are reported as odds ratios, including 95% confidence intervals (not corrected for multiple testing) and p-values corrected for multiple testing. No results were significant after multiple testing correction.

RQ1a: What feeding patterns exist among cat guardians?		
**Variable item**	**Status cf. reference category**	**Statistics**
**Likelihood among vegan guardians of feeding vegan cat food**		
**HUMAN DEMOGRAPHICS**		
Age: 30-39	No trend cf. ages 18-29	OR = +109% (CI: [-0.2%, +337%], p>0.9999)
Age: 40-49	Trend cf. ages 18-29	OR = +204% (CI: [+37%, +575%], p=0.2371)
Gender: male	Trend cf. females	OR = +112% (CI: [+2%, +341%], p>0.9999)
Region: Oceania	No trend cf. UK residents	OR = +147% (CI: [-32%, +805%], p>0.9999)
Region: North America	Trend cf. UK residents	OR = +405% (CI: [+88%, +1256%], p=0.0519)
Region: other European	No trend cf. UK residents	OR = +104% (CI: [-0.1%, +316%], p>0.9999)
Income: medium	No trend cf. low income	OR = -24% (CI: [-62%, +51%], p>0.9999)
Income: high	No trend cf. low income	OR = -37% (CI: [-78%, +83%], p>0.9999)
Residence: equally urban/rural	No trend cf. urban	OR = +46% (CI: [-30%, +205%], p>0.9999)
Residence: rural	No trend cf. urban	OR = +33% (CI: [-32%, +160%], p>0.9999)
No/minimal effect: education, pet/vet industry		

**CAT DEMOGRAPHICS**		
Age: 5-9	No trend cf. ages 0-4	OR = -17% (CI: [-55%, +52%], p>0.9999)
Age: 10-14	No trend cf. ages 0-4	OR = -49% (CI: [-75%, +7%], p>0.9999)
Age: 15-24	Trend cf. ages 0-4	OR = -83% (CI: [-96%, -24%], p=0.7099)
Medical diet	Trend cf. not on medical diet	OR = -86% (CI: [-96%, -44%], p=0.1988)
Sex/neutered: F, sexually intact	No trend cf. F, spayed	OR = +179% (CI: [-84%, +4735%], p>0.9999)
Sex/neutered: M, sexually intact	No trend cf. F, spayed	OR = -44% (CI: [-95%, +606%], p>0.9999)
Sex/neutered: M, castrated	No trend cf. F, spayed	OR = -6% (CI: [-45%, +61%), p>0.9999)
Habitat: indoor and outdoor	No trend cf. mostly indoor	OR = -5% (CI: [-48%, +74%), p>0.9999)
Habitat: mostly outdoor	No trend cf. mostly indoor	OR = +147% (CI: [-43%, +980%], p>0.9999)
**Likelihood among guardians feeding meat, of feeding raw meat**		
**HUMAN DEMOGRAPHICS**		
Region: North America	Trend cf. UK residents	OR = +322% (CI: [+56%, +1045%], p=0.2280)
Region: Oceania	No trend cf. UK residents	OR = +224% (CI: [-6%, +1017%], p>0.9999)
Region: other European	No trend cf. UK residents	OR = +47% (CI: [-39%, +253%], p>0.9999)
No/minimal effect: all other variables		
**CAT DEMOGRAPHICS**		
Age: 5-9	No trend cf. ages 0-4	OR = -2% (CI: [-49%, +89%], p>0.9999)
Age: 10-14	No trend cf. ages 0-4	OR = -42% (CI: [-73%, +27%], p>0.9999)
Age: 15-24	No trend cf. ages 0-4	OR = -53% (CI: [-85%, +48%], p>0.9999)
Sex/neutered: F, sexually intact	No trend cf. F, spayed	OR = +141% (CI: [-72%, +1967%], p>0.9999)
Sex/neutered: M, sexually intact	Trend cf. F, spayed	OR = +433% (CI: [+3%, +2641%], p>0.9999)
Sex/neutered: M, castrated	Trend cf. F, spayed	OR = +77% (CI: [+0.2%, +213%], p>0.9999)
Medical diet	No trend cf. no medical diet	OR = -57% (CI: [-88%, +49%], p>0.9999)
Habitat: indoor and outdoor	No trend cf. mostly indoor	OR = -44% (CI: [-70%, +6%], p>0.9999)
Habitat: mostly outdoor	No trend cf. mostly indoor	OR = -37% (CI: [-86%, +190%], p>0.9999)

Table S1b. Key statistics relating to RQ1b (current purchasing determinants) and Figure 7.

Note: Starred items in the first column relate to Figure S2. Effects are reported including 95% confidence intervals (not corrected for multiple testing) and p-values corrected for multiple testing. Significant results (after multiple testing correction) are highlighted. P-values are not provided in cases of explorative analyses.

RQ1b: What factors do cat guardians find important when choosing cat diets?			
**Purchasing determinant category / item***	**Variable item**	**Status cf. reference category**	**Statistics**
**HUMAN DEMOGRAPHICS**			
**Personal Values**	**Diet: reducetarian**	**Effect cf. omnivores**	**Estimate = +0.12 (CI: [+0.08, +0.17], p=0.0001)**
**Personal Values**	**Diet: pescatarian**	**Effect cf. omnivores**	**Estimate = +0.17 (CI: [+0.09, +0.26], p=0.0100)**
**Personal Values**	**Diet: vegetarian**	**Effect cf. omnivores**	**Estimate = +0.25 (CI: [+0.19, +0.32], p<0.0001)**
**Personal Values**	**Diet: vegan**	**Effect cf. omnivores**	**Estimate = +0.41 (CI: [+0.37, +0.46], p<0.0001)**
**Personal Focus**	**Age: 50-59**	**Effect cf. ages 18-29**	**Estimate = -0.11 (CI: [-0.16, -0.05], p=0.0269)**
**Personal Focus**	**Age: 70+**	**Effect cf. ages 18-29**	**Estimate = -0.14 (CI: [-0.21, -0.07], p=0.0128)**
Pet Focus I	Pet/vet industry	Trend cf. non pet/vet ind.	Estimate = +0.09 (CI: [+0.04, +0.14], p=0.1855)
Diet quality*	Pet/vet industry	Explorative tendency cf. non pet/vet ind.	OR = +162% (CI: [+62%, +323%])
Pet Focus II	Pet/vet industry	Trend cf. non pet/vet ind.	Estimate = +0.07 (CI: [+0.02, +0.12], p>0.9999)
Freshness*	Pet/vet industry	Explorative tendency cf. non pet/vet ind.	OR = +93% (CI: [+21%, +208%])
Personal Values	Pet/vet industry	Trend cf. non pet/vet ind.	Estimate = +0.11 (CI: [+0.04, +0.18], p=0.4161)
Food animal concerns*	Pet/vet industry	Explorative tendency cf. non pet/vet ind.	OR = +137% (CI: [+56%, +258%])
**Pet Focus II**	**Region: North America**	**Effect cf. UK residents**	**Estimate = +0.16 (CI: [+0.09, +0.23], p=0.0050)**
**Personal Values**	**Region: North America**	**Effect cf. UK residents**	**Estimate = +0.29 (CI: [+0.19, +0.38], p<0.0001)**
**Personal Values**	**Region: other European**	**Effect cf. UK residents**	**Estimate = +0.20 (CI: [+0.14, +0.25], p<0.0001)**
Pet Focus I	Educ.: high school	Trend cf. UK residents	Estimate = -0.13 (CI: [-0.21, -0.05], p=0.3067)
No/minimal effect: gender, income, residence			

CAT DEMOGRAPHICS			
**Pet Focus II**	**Diet: raw meat**	**Effect cf. conventional meat**	**Estimate = +0.22 (CI: [+0.14, +0.29], p<0.0001)**
Personal Focus	Diet: raw meat	No trend cf. conventional meat	Estimate = -0.06 (CI: [-0.13, +0.01], p>0.9999)
Personal Values	Diet: raw meat	Trend cf. conventional meat	Estimate = +0.15 (CI: [+0.06, +0.25], p=0.2942)
**Personal Focus**	**Diet: vegan**	**Effect cf. conventional meat**	**Estimate = -0.13 (CI: [-0.18, -0.08], p<0.0001)**
**Personal Values**	**Diet: vegan**	**Effect cf. conventional meat**	**Estimate = +0.40 (CI: [+0.33, +0.46], p<0.0001)**
Pet Focus II	Age: 15-24	Trend cf. ages 0-4	Estimate = -0.07 (CI: [-0.13, -0.01], p>0.9999)
Pet Focus I	Habitat: mostly outdoor	No trend cf. mostly indoor	Estimate = -0.07 (CI: [-0.15, +0.004], p>0.9999)
Pet Focus II	Habitat: mostly outdoor	No trend cf. mostly indoor	Estimate = -0.07 (CI: [-0.15, +0.02], p>0.9999)
Personal Values	Habitat: mostly outdoor	No trend cf. mostly indoor	Estimate = -0.10 (CI: [-0.20, +0.005], p>0.9999)
Pet Focus II	Sex/neutered: F, sexually intact	No trend cf. F, spayed	Estimate = +0.10 (CI: [-0.04, +0.24], p>0.9999)
Pet Focus II	Sex/neutered: M, sexually intact	No trend cf. F, spayed	Estimate = +0.03 (CI: [-0.11, +0.17], p>0.9999)
Pet Focus II	Sex/neutered: M, castrated	No trend cf. F, spayed	Estimate = +0.01 (CI: [-0.02, +0.04], p>0.9999)
No/minimal effect: medical diet			

Table S2a. Key statistics relating to RQ2a (acceptance of alternative cat foods) and Figures 9 and 10.

Note: Effects are reported as odds ratios, including 95% confidence intervals (not corrected for multiple testing) and p-values corrected for multiple testing. Significant results (after multiple testing correction) are highlighted.

RQ2a: What proportion of guardians feeding conventional or raw meat-based cat diets would realistically accept more sustainable alternatives?			
**Alternative diet**	**Variable item**	**Status cf. reference category**	**Statistics**
**HUMAN DEMOGRAPHICS**			
**100% non-animal: plant-based**	**Diet: reducetarian**	**Effect cf. omnivores**	**OR = +756% (CI: [+222%, +2178%], p=0.0052)**
**100% non-animal: plant-based**	**Diet: pescatarian**	**Effect cf. omnivores**	**OR = +1379% (CI: [+377%, +4486%], p=0.0010)**
**100% non-animal: plant-based**	**Diet: vegetarian**	**Effect cf. omnivores**	**OR = +2565% (CI: [+906%, +6961%], p<0.0001)**
**100% non-animal: fungi-based**	**Diet: vegetarian**	**Effect cf. omnivores**	**OR = +726% (CI: [+301%, +1602%], p<0.0001)**
**100% non-animal: algae-based**	**Diet: vegetarian**	**Effect cf. omnivores**	**OR = +669% (CI: [+260%, +1540%], p<0.0001)**
**Vegetarian**	**Diet: vegetarian**	**Effect cf. omnivores**	**OR = +300% (CI: [+139%, +559%], p<0.0001)**
**Cultivated meat**	**Diet: vegetarian**	**Effect cf. omnivores**	**OR = +157% (CI: [+72%, +283%], p=0.0012)**
**Insect-based**	**Diet: vegetarian**	**Effect cf. omnivores**	**OR = +235% (CI: [+103%, +451%], p=0.0007)**
**100% non-animal: plant-based**	**Diet: vegan**	**Effect cf. omnivores**	**OR = +18332% (CI: [+7165%, +46664%], p<0.0001)**
**100% non-animal: fungi-based**	**Diet: vegan**	**Effect cf. omnivores**	**OR = +1106% (CI: [+527%, +2218%], p<0.0001)**
**100% non-animal: algae-based**	**Diet: vegan**	**Effect cf. omnivores**	**OR = +1226% (CI: [+574%, +2510%], p<0.0001)**
**Cultivated meat**	**Diet: vegan**	**Effect cf. omnivores**	**OR = +126% (CI: [+60%, +219%], p=0.0010)**
**Insect-based**	**Diet: vegan**	**Effect cf. omnivores**	**OR = +154% (CI: [+61%, +300%], p=0.0192)**
**Cultivated meat**	**Age: 40-49**	**Effect cf. ages 18-29**	**OR = -64% (CI: [-78%, -42%], p=0.0101)**
**Insect-based**	**Age: 40-49**	**Effect cf. ages 18-29**	**OR = -73% (CI: [-86%, -48%], p=0.0244)**
**Cultivated meat**	**Age: 50-59**	**Effect cf. ages 18-29**	**OR = -68% (CI: [-80%, -47%], p=0.0017)**
100% non-animal: plant-based	Age: 70+	Trend cf. ages 18-29	OR = -87% (CI: [-96%, -53%], p=0.5168)

100% non-animal: fungi-based	Age: 70+	Trend cf. ages 18-29	OR = -70% (CI: [-90%, -12%], p>0.9999)
100% non-animal: algae-based	Age: 70+	Trend cf. ages 18-29	OR = -78% (CI: [-94%, -19%], p>0.9999)
Vegetarian	Age: 70+	No trend cf. ages 18-29	OR = -61% (CI: [-85%, +1%], p>0.9999)
**Cultivated meat**	**Age: 70+**	**Effect cf. ages 18-29**	**OR = -78% (CI: [-89%, -57%], p=0.0022)**
100% non-animal: plant-based	Educ.: high school	No trend cf. those with doctorate	OR = -52% (CI: [-81%, +18%], p>0.9999)
100% non-animal: algae-based	Educ.: high school	Trend cf. those with doctorate	OR = -71% (CI: [-90%, -13%], p>0.9999)
Vegetarian	Educ.: high school	No trend cf. those with doctorate	OR = -59% (CI: [-84%, +3%], p>0.9999)
Cultivated meat	Educ.: high school	No trend cf. those with doctorate	OR = -57% (CI: [-79%, -12%], p>0.9999)
Insect-based	Educ.: high school	Trend cf. those with doctorate	OR = -79% (CI: [-91%, -51%], p=0.1093)
100% non-animal: plant-based	Pet/vet industry	No trend cf. non pet/vet ind.	OR = -39% (CI: [-68%, +16%], p>0.9999)
Vegetarian	Pet/vet industry	Trend cf. non pet/vet ind.	OR = -64% (CI: [-83%, -23%], p>0.9999)
Cultivated meat	Pet/vet industry	No trend cf. non pet/vet ind.	OR = +37% (CI: [-11%, +112%], p>0.9999)
Insect-based	Pet/vet industry	No trend cf. non pet/vet ind.	OR = +40% (CI: [-17%, +136%], p>0.9999)
**100% non-animal: plant-based**	**Region: other European**	**Effect cf. UK residents**	**OR = +837% (CI: [+490%, +1387%], p<0.0001)**
100% non-animal: plant-based	Region: North America	No trend cf. UK residents	OR = +114% (CI: [-7%%, +392%], p>0.9999)
100% non-animal: plant-based	Region: Oceania	Trend cf. UK residents	OR = +175% (CI: [+18%, +542%], p>0.9999)
No/minimal effect: gender, income, residence			
**CAT DEMOGRAPHICS**			
100% non-animal: plant-based	Diet: raw meat	No trend cf. conventional meat	OR = -51% (CI: [-79%, +17%], p>0.9999)
100% non-animal: fungi-based	Diet: raw meat	No trend cf. conventional meat	OR = -55% (CI: [-86%, +49%], p>0.9999)
100% non-animal: algae-based	Diet: raw meat	No trend cf. conventional meat	OR = -68% (CI: [-92%, +35%], p>0.9999)
Vegetarian	Diet: raw meat	Trend cf. conventional meat	OR = -79% (CI: [-95%, -14%], p>0.9999)
No/minimal effect: all other cat demographic variables			

Table S2b. Key statistics relating to RQ2b (essential characteristics of alternative cat foods) and Figure 11.

Note: Effects are reported as odds ratios, including 95% confidence intervals (not corrected for multiple testing) and p-values corrected for multiple testing. Significant results (after multiple testing correction) are highlighted.

RQ2b: What are the essential characteristics of alternative cat foods?			
**Essential category / item***	**Variable item**	**Status cf. reference category**	**Statistics**
**HUMAN DEMOGRAPHICS**			
**Personal Values**	**Diet: reducetarian**	**Effect cf. omnivores**	**OR = +94% (CI: [+36%, +176%], p=0.0404)**
**Personal Values**	**Diet: pescatarian**	**Effect cf. omnivores**	**OR = +207% (CI: [+78%, +429%], p=0.0092)**
**Pet Focus I**	**Diet: vegetarian**	**Effect cf. omnivores**	**OR = +326% (CI: [+185%, +537%], p<0.0001)**
**Pet Focus II**	**Diet: vegetarian**	**Effect cf. omnivores**	**OR = +290% (CI: [+159%, +486%], p<0.0001)**
**Personal Focus**	**Diet: vegetarian**	**Effect cf. omnivores**	**OR = +203% (CI: [+102%, +353%], p<0.0001)**
**Personal Values**	**Diet: vegetarian**	**Effect cf. omnivores**	**OR = 474% (CI: [+279%, +769%], p<0.0001)**
**Pet Focus I**	**Diet: vegan**	**Effect cf. omnivores**	**OR = +1196% (CI: [+756%, +1863%], p<0.0001)**
**Pet Focus II**	**Diet: vegan**	**Effect cf. omnivores**	**OR = +345% (CI: [+213%, +531%], p<0.0001)**
**Personal Focus**	**Diet: vegan**	**Effect cf. omnivores**	**OR = +311% (CI: [+191%, +479%], p<0.0001)**
**Personal Values**	**Diet: vegan**	**Effect cf. omnivores**	**OR = +793% (CI: [+519%, +1187%], p<0.0001)**
**Pet Focus I**	**Age: 60-69**	**Effect cf. ages 18-29**	**OR = -63% (CI: [-78%, -39%], p=0.0197)**
**Pet Focus I**	**Age: 70+**	**Effect cf. ages 18-29**	**OR = -79% (CI: [-89%, -60%], p=0.0003)**
**Personal Focus**	**Age: 70+**	**Effect cf. ages 18-29**	**OR = -72% (CI: [-85%, -46%], p=0.0268)**
**Personal Values**	**Age: 70+**	**Effect cf. ages 18-29**	**OR = -74% (CI: [-87%, -48%], p=0.0250)**
Pet Focus I	Educ.: high school	Trend cf. those with doctorate	OR = -60% (CI: [-81%, -17%], p>0.9999)
Personal Values	Educ.: high school	Trend cf. those with doctorate	OR = -72% (CI: [-86%, -42%], p=0.0871)
**Pet Focus I**	**Region: other European**	**Effect cf. UK residents**	**OR = +433% (CI: [+236%, +744%], p<0.0001)**
**Pet Focus II**	**Region: other European**	**Effect cf. UK residents**	**OR = +155% (CI: [+72%, +277%], p=0.0005)**
**Personal Values**	**Region: other European**	**Effect cf. UK residents**	**OR = +230% (CI: [+122%, +389%], p<0.0001)**
Personal Values	Gender: male	No trend cf. females	OR = -40% (CI: [-64%, +2], p>0.9999)
No/minimal effect: pet/vet industry, income, residence			

CAT DEMOGRAPHICS			
Pet Focus II	Diet: raw meat	No trend cf. conventional meat	OR = +54% (CI: [-10%, +165%], p>0.9999)
Personal Values	Diet: raw meat	No trend cf. conventional meat	OR = +59% (CI: [-7%, +172%], p>0.9999)
Pet Focus I	Age: 10-14	Trend cf. ages 0-4	OR = +73% (CI: [+21%, +147%], p=0.4188)
Personal Focus	Age: 10-14	Trend cf. ages 0-4	OR = +56% (CI: [+10%, +122%], p>0.9999)
Personal Values	Age: 10-14	No trend cf. ages 0-4	OR = +38% (CI: [-4%, +98%], p>0.9999)
Pet Focus I	Age: 15-24	No trend cf. ages 0-4	OR = +28% (CI: [-20%, +105%], p>0.9999)
Personal Focus	Age: 15-24	No trend cf. ages 0-4	OR = +19% (CI: [-26%, +92%], p>0.9999)
Personal Values	Age: 15-24	No trend cf. ages 0-4	OR = +14% (CI: [-30%, +86%], p>0.9999)
No/minimal effect: medical diet, sex/neuter status, habitat			

Table S3. Key statistics relating to RQ3 (information sources about cat diets) and Figure 13 from the main paper.

Note: Effects are reported as odds ratios, including 95% confidence intervals (not corrected for multiple testing) and p-values corrected for multiple testing. Significant results (after multiple testing correction) are highlighted. The starred item in the first column relates to Figure S7; for this effect, no multiple testing correction was applied due to the exploratory nature of this analysis. P-values are not provided in cases of explorative analyses.

**RQ3: Where do cat guardians source information about cat diets from?**			
**Source category / item***	**Variable item**	**Status cf. reference category**	**Statistics**
**HUMAN DEMOGRAPHICS**			
**Media/Literature**	**Diet: vegetarian**	**Effect cf. omnivores**	**OR = +121% (CI: [+47%, +231%], p=0.0235)**
**Product-Specific**	**Diet: vegan**	**Effect cf. omnivores**	**OR = +76% (CI: [+33%, +134%], p=0.0156)**
**Media/Literature**	**Diet: vegan**	**Effect cf. omnivores**	**OR = +333% (CI: [+219%, +488%], p<0.0001)**
**Social Media**	**Diet: vegan**	**Effect cf. omnivores**	**OR = +183% (CI: [+98%, +303%], p<0.0001)**
Product-Specific	Age: 70+	Trend cf. omnivores	OR = -53% (CI: [-72%, -18%], p>0.9999)
Media/Literature	Age: 70+	Trend cf. omnivores	OR = -59% (CI: [-78%, -24%], p=0.8055)
Social Media	Age: 70+	No trend cf. omnivores	OR = -36% (CI: [-69%, +32%], p>0.9999)
Product-Specific	Educ.: high school	Trend cf. those with doctorate	OR = -54% (CI: [-75%, -13%], p>0.9999)
Vet/Pet Care	Educ.: high school	Trend cf. those with doctorate	OR = -66% (CI: [-82%, -34%], p=0.2390)
Social Media	Educ.: high school	No trend cf. those with doctorate	OR = +42% (CI: [-35%, +208%], p>0.9999)
Vet/Pet Care	Pet/vet industry	Trend cf. non pet/vet ind.	OR = +82% (CI: [+20%, +176%], p=0.8119)
Media/Literature	Pet/vet industry	Trend cf. non pet/vet ind.	OR = +86% (CI: [+24%, +179%], p=0.4981)
**Media/Literature**	**Region: other European**	**Effect cf. UK residents**	**OR = +122% (CI: [+58%, +214%], p=0.0010)**
**Media/Literature**	**Region: North America**	**Effect cf. UK residents**	**OR = +208% (CI: [+77%, +436%], p=0.0129)**
Vet/Pet Care	Income: high	Trend those on low income	OR = +64% (CI: [+3%, +163%], p>0.9999)
Media/Literature	Income: high	No trend those on low income	OR = +56% (CI: [-4%, +152%], p>0.9999)
Social Media	Income: high	No trend cf. those on low income	OR = +38% (CI: [-21%, +140%], p>0.9999)
No/minimal effect: residence, gender			

CAT DEMOGRAPHICS			
**Media/Literature**	**Diet: raw meat**	**Effect cf. conventional meat**	**OR = +210% (CI: [+81%, +431%], p=0.0073)**
**Social Media**	**Diet: raw meat**	**Effect cf. conventional meat**	**OR = +212% (CI: [+78%, +449%], p=0.0138)**
**Media/Literature**	**Diet: vegan**	**Effect cf. conventional meat**	**OR = +333% (CI: [+190%, +546%], p<0.0001)**
**Social Media**	**Diet: vegan**	**Effect cf. conventional meat**	**OR = +466% (CI: [+280%, +742%], p<0.0001)**
**Vet/Pet Care**	**Medical diet**	**Effect cf. no medical diet**	**OR = +261% (CI: [+113%, +484%], p<0.0001)**
Veterinarians*	Medical diet	Explorative tendency cf. no medical diet	OR = +307% (CI: [+155%, +549%])
Vet/Pet Care	Sex/neutered: F, sexually intact	No trend cf. F, spayed	OR = -75% (CI: [-94%, +0.004%], p>0.9999)
Vet/Pet Care	Sex/neutered: M, sexually intact	No trend cf. F, spayed	OR = -57% (CI: [-86%, +38%], p>0.9999)
Vet/Pet Care	Sex/neutered: M, castrated	No trend cf. F, spayed	OR = +14% (CI: [-10%, +43%], p>0.9999)
Product-Specific	Habitat: indoor and outdoor	No trend cf. mostly indoor	OR = -10% (CI: [-29%, +15%], p>0.9999)
Vet/Pet Care	Habitat: indoor and outdoor	No trend cf. mostly indoor	OR = -17% (CI: [-35%, +7%], p>0.9999)
Media/Literature	Habitat: indoor and outdoor	No trend cf. mostly indoor	OR = -4% (CI: [-27%, +25%], p>0.9999)
Social Media	Habitat: indoor and outdoor	No trend cf. mostly indoor	OR = -22% (CI: [-44%, +7%], p>0.9999)
Product-Specific	Habitat: mostly outdoor	No trend cf. mostly indoor	OR = -22% (CI: [-57%, +41%], p>0.9999)
Vet/Pet Care	Habitat: mostly outdoor	No trend cf. mostly indoor	OR = -29% (CI: [-62%, +33%], p>0.9999)
Media/Literature	Habitat: mostly outdoor	Trend cf. mostly indoor	OR = -60% (CI: [-80%, -17%], p>0.9999)
Social Media	Habitat: mostly outdoor	No trend cf. mostly indoor	OR = -21% (CI: [-63%; +69%], p>0.9999)
No/minimal effect: age			
