# Supplementary material for: Consumer Acceptance of Sustainable Cat Diets: A Survey of 1380 Cat Guardians
Source: Animals (Basel). 2025 Oct 15;15(20):2984. doi: 10.3390/ani15202984 (PMC12560941; doi:10.3390/ani15202984)
Supplement: Supplementary file 1 [file animals-15-02984-s001.zip › animals-3806110-Supplementary Figures.pdf]

Supplementary Figures

Part 1 – Current diets

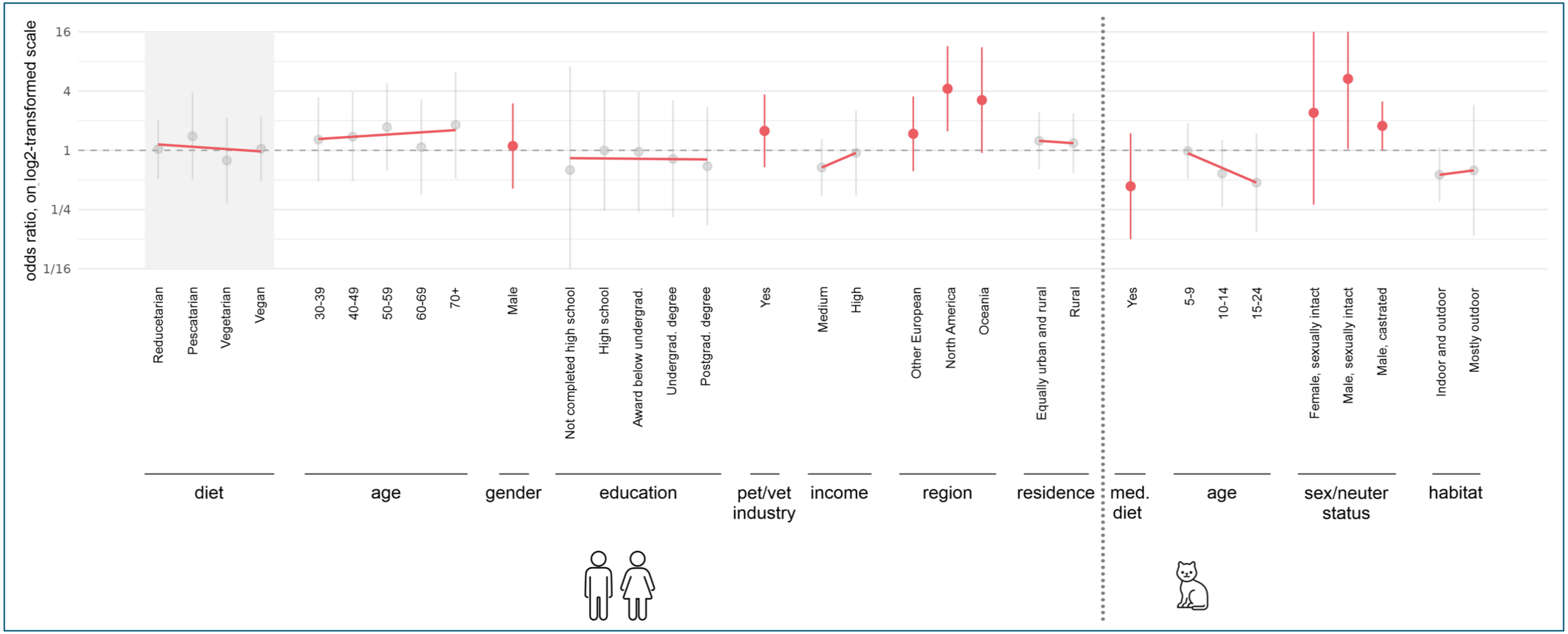

Figure S1. Logistic regression results on the associations between human/cat demographic characteristics and the likelihood of currently feeding raw meat-based diets to cats (as opposed to conventional meat-based diets), relative to the reference characteristics for each group.

Note: The reference characteristics for these humans and cats were respectively: (human) aged 18–29, female, doctoral degree, not in pet/vet industry, low income, UK- and urban-based; (cat) no medical diet, aged 0–4, female, spayed, mostly indoors. Effects are depicted as odds ratios, including 95% confidence intervals (not corrected for multiple testing). No effect was significant after correction for multiple testing. Individual estimated effects of ordinal variables are grayed out in favor of an additional linear trend line. Effects of human diet are highlighted through a gray-shaded area to reflect their separate estimation scheme (i.e., these effects were estimated while not controlling for further human demographic variables) as outlined in section 2.2.

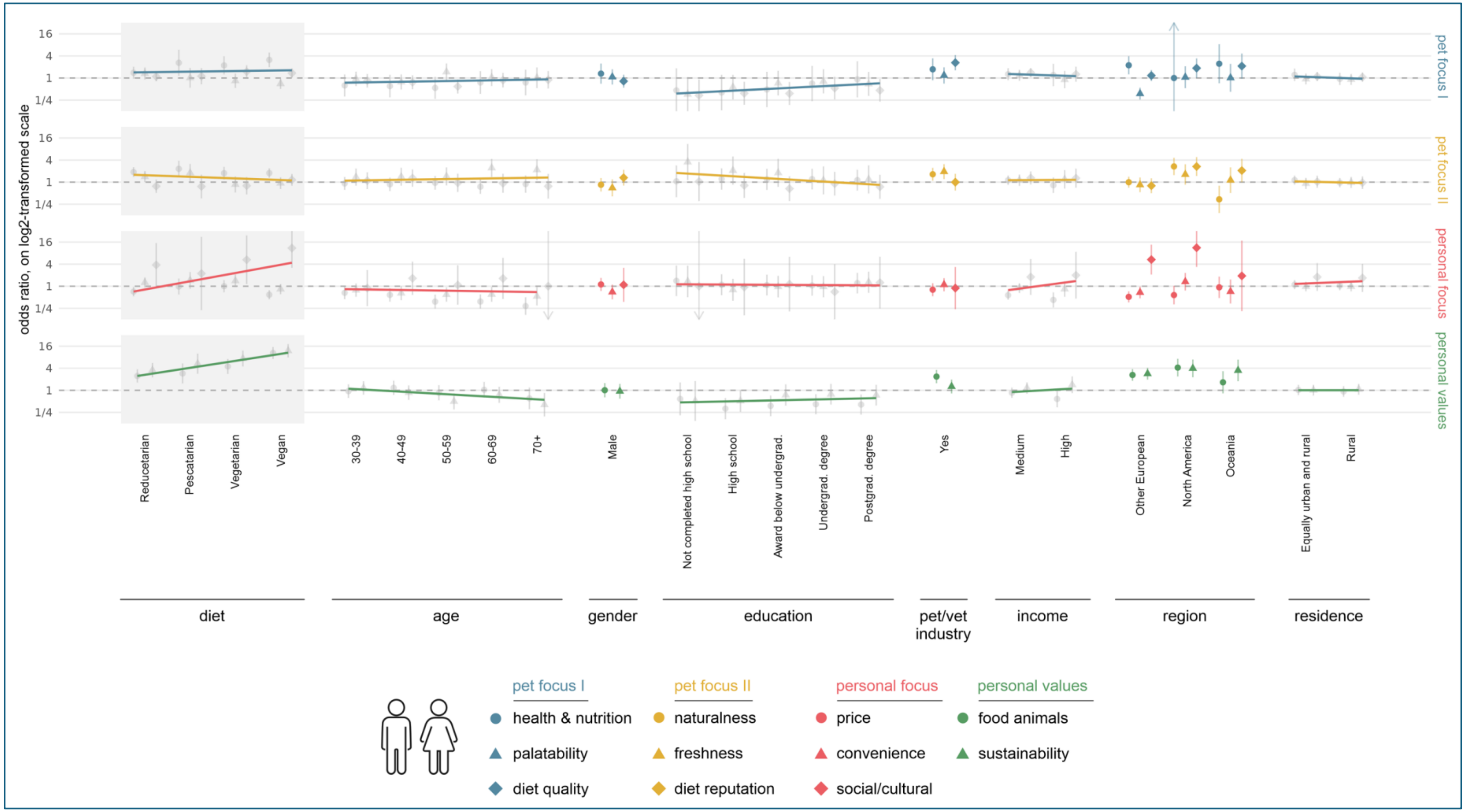

Figure S2. Logistic regression results on the associations between human demographic characteristics and current cat food purchasing determinants, relative to the reference characteristics for each group.

Note: This figure relates to Figure 7 from the main paper. It illustrates the results regarding the individual items that comprise the right y-axis categories. The reference characteristics for these humans were: omnivore, aged 18–29, female, doctoral degree, not in pet/vet industry, low income, UK-based, and urban-based. Effects are depicted as odds ratios, including 95% confidence intervals. Due to the exploratory character of this analysis, no multiple testing correction was applied and statistical significance was not investigated. Individual estimated effects of ordinal variables are grayed out in favor of an additional linear trend line. Effects of human diet are highlighted through gray-shaded areas to reflect their separate estimation scheme (i.e., these effects were estimated while not controlling for further human demographic variables) as outlined in section 2.2.

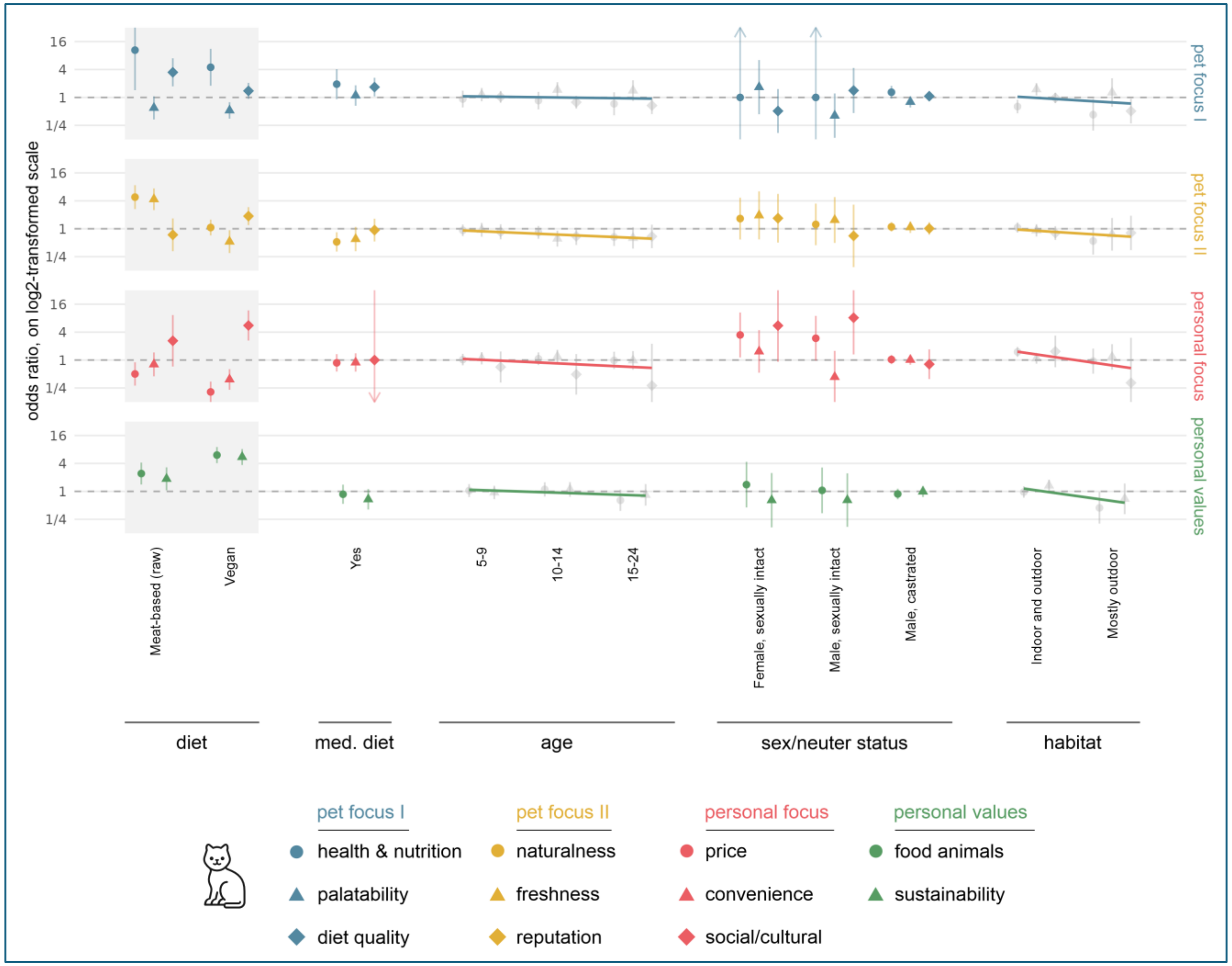

Figure S3. Logistic regression results on the associations between cat demographic characteristics and current cat food purchasing determinants, relative to the reference characteristics for each group.

<u>Note</u>: This figure relates to Figure 7 from the main paper. It illustrates the results regarding the individual items that comprise the right y-axis categories. The reference characteristics for these cats were: meat-based (conventional); no medical diet; aged 0–4; female, spayed; and mostly indoors. Effects are depicted as odds ratios, including 95% confidence intervals. Due to the exploratory character of this analysis, no multiple testing correction was applied and statistical significance was not investigated. Individual estimated effects of ordinal variables are grayed out in favor of an additional linear trend line. Effects of cat diet are highlighted through gray-shaded areas to reflect their separate estimation scheme (i.e., these effects were estimated while not controlling for further human demographic variables) as outlined in section 2.2.

Part 2 – Alternative diets

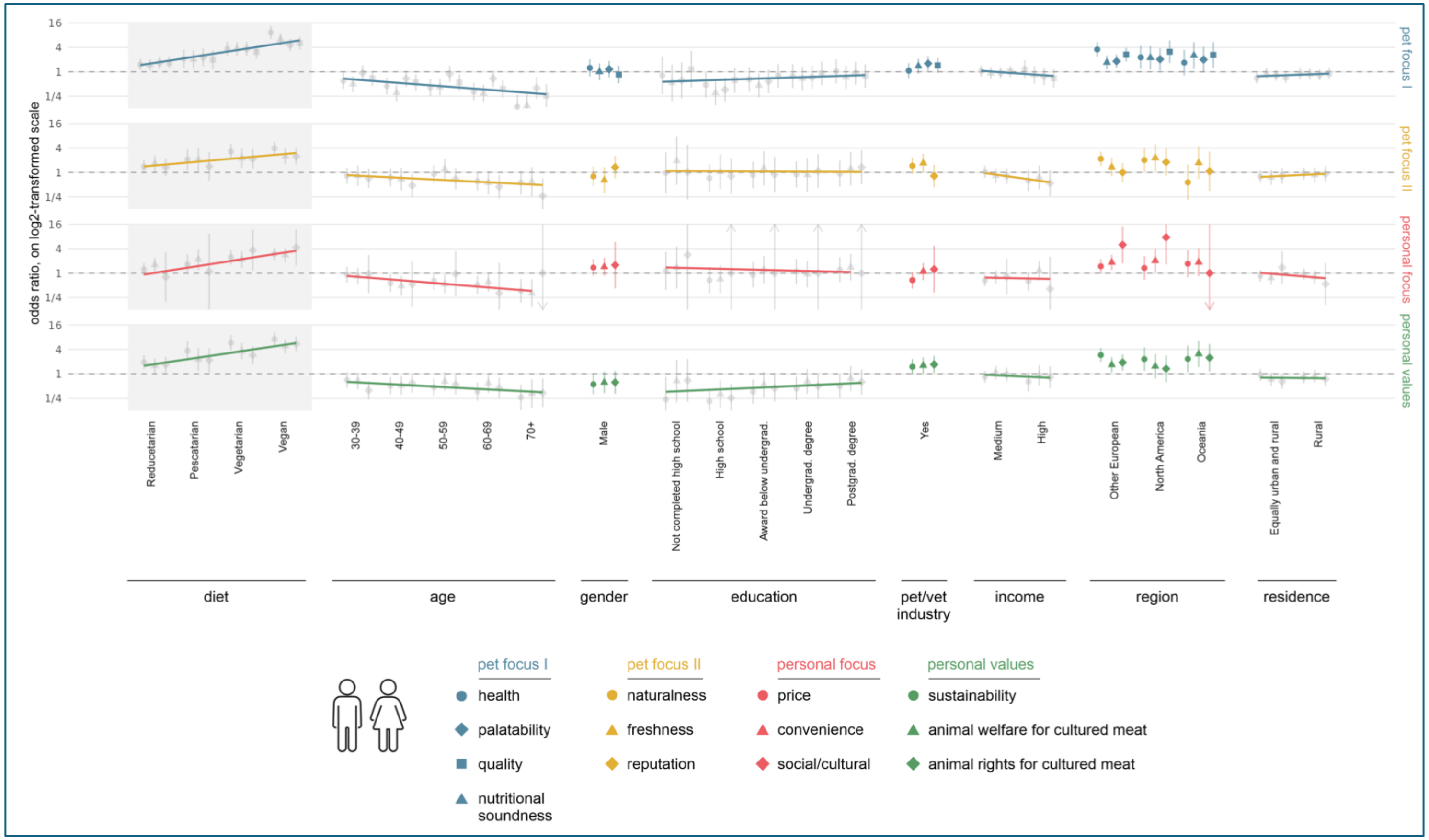

Figure S4. Logistic regression results on the associations between human demographic characteristics and characteristics of more sustainable cat diets considered essential, among guardians currently feeding meat-based cat food (raw or conventional), relative to the reference characteristics for each group.

Note: This figure relates to Figure 11 from the main paper and illustrates the results regarding the individual items that comprise the right y-axis categories. The reference characteristics for these humans were: omnivore, aged 18–29, female, doctoral degree, not in pet/vet industry, low income, UK-based, and urban-based. Effects are depicted as odds ratios, including 95% confidence intervals. Due to the exploratory character of this analysis, no multiple testing correction was applied and statistical significance was not investigated. Individual estimated effects of ordinal variables are grayed out in favor of an additional linear trend line. Effects of human diet are highlighted through gray-shaded areas to reflect their separate estimation scheme (i.e., these effects were estimated while not controlling for further human demographic variables) as outlined in section 2.2.

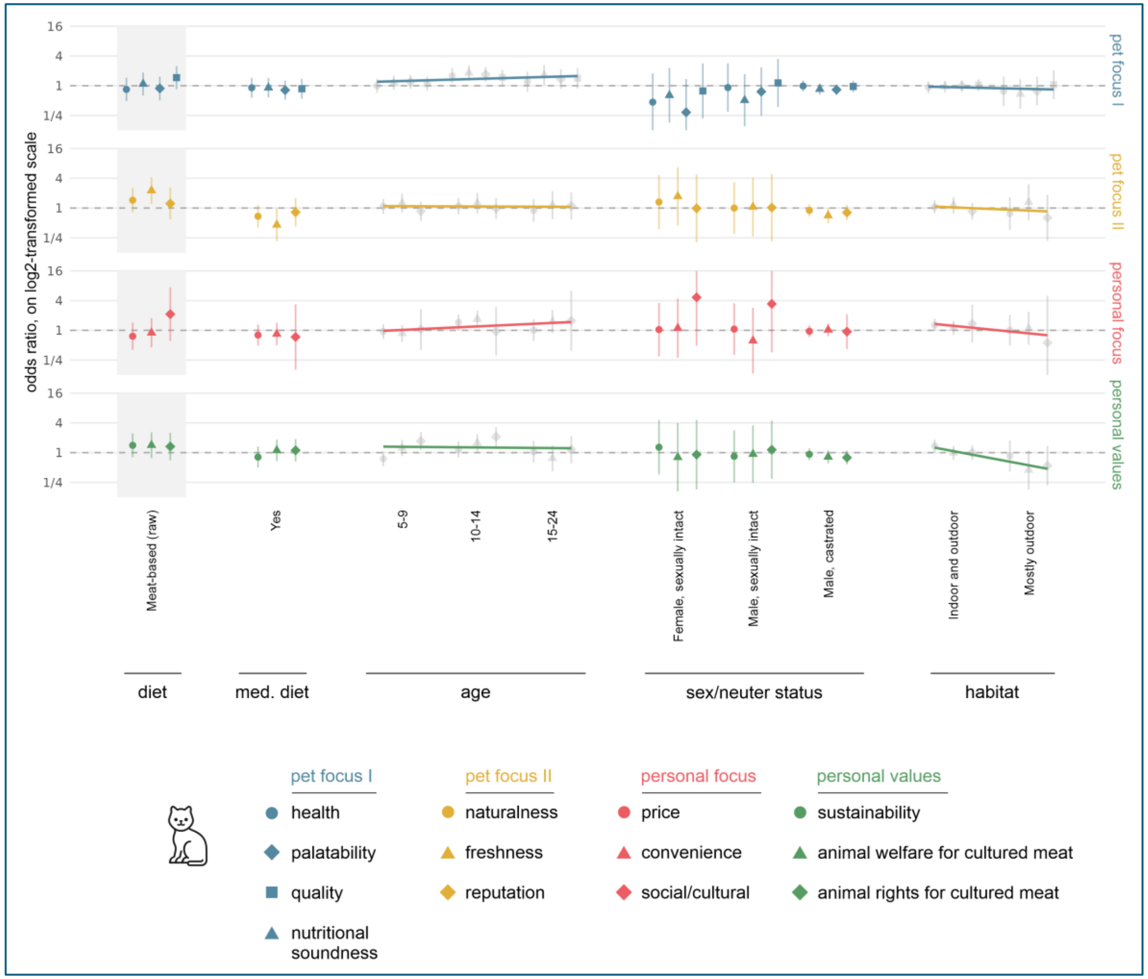

Figure S5. Logistic regression results on the associations between cat demographic characteristics and characteristics of more sustainable cat diets considered essential, among guardians currently feeding meat-based cat food (raw or conventional), relative to the reference characteristics for each group.

Note: This figure relates to Figure 11 from the main paper and illustrates the results regarding the individual items that comprise the right y-axis categories. The reference characteristics for these cats were: meat-based (conventional); no medical diet; aged 0–4; female, spayed; and mostly indoors. Effects are depicted as odds ratios, including 95% confidence intervals. Due to the exploratory character of this analysis, no multiple testing correction was applied and statistical significance was not investigated. Individual estimated effects of ordinal variables are grayed out in favor of an additional linear trend line. Effects of cat diet are highlighted through gray-shaded areas to reflect their separate estimation scheme (i.e., these effects were estimated while not controlling for further human demographic variables) as outlined in section 2.2.

Part 3 – Information sources

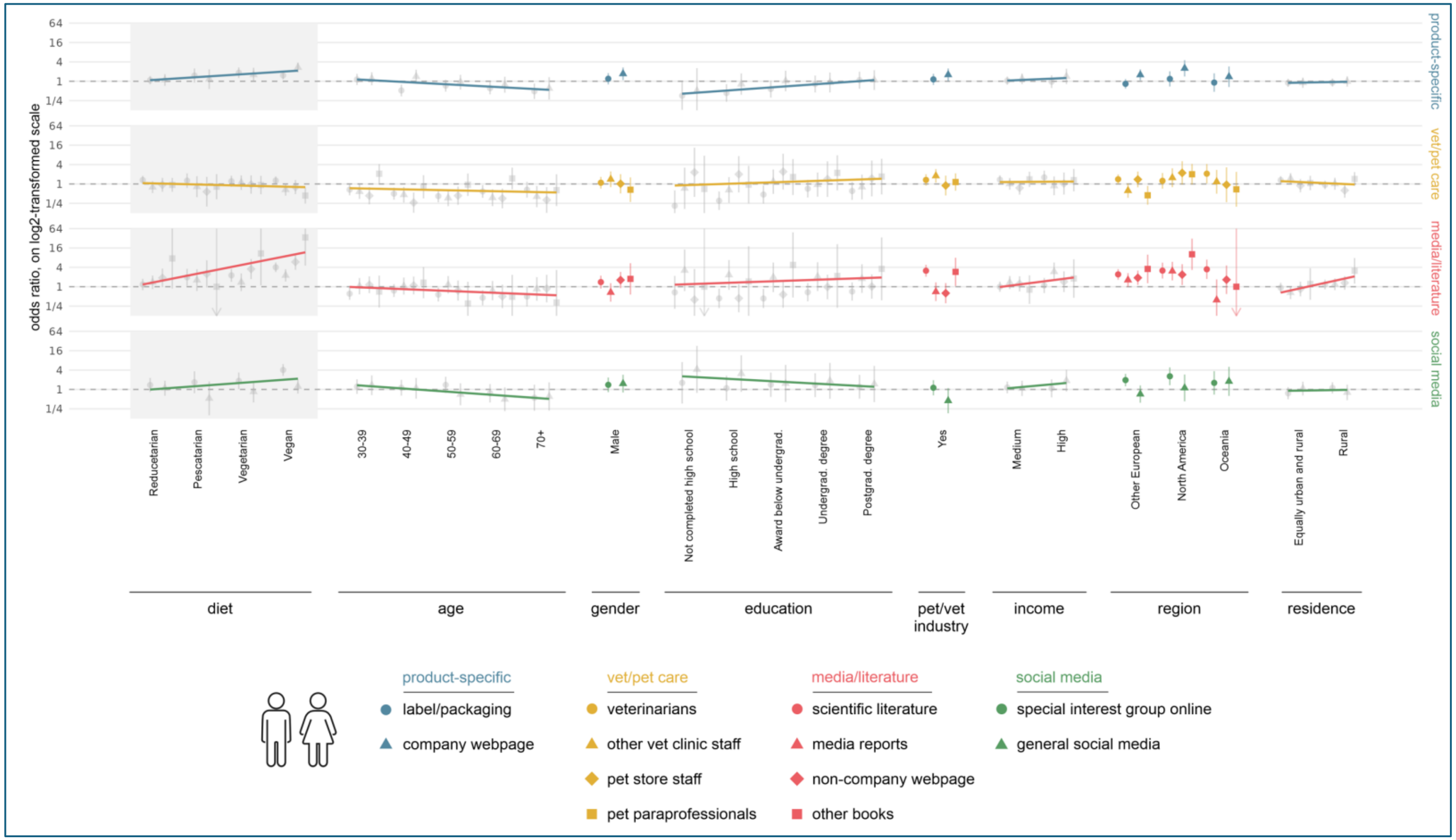

Figure S6. Logistic regression results on the associations between human demographic characteristics and dietary information sources, relative to the reference characteristics for each group.

Note: This figure relates to Figure 13 from the main paper and illustrates the results regarding the individual items that comprise the right y-axis categories. The reference characteristics for these humans were: omnivore, aged 18–29, female, doctoral degree, not in pet/vet industry, low income, UK-based, and urban-based. Effects are depicted as odds ratios, including 95% confidence intervals. Due to the exploratory character of this analysis, no multiple testing correction was applied and statistical significance was not investigated. Individual estimated effects of ordinal variables are grayed out in favor of an additional linear trend line. Effects of human diet are highlighted through gray-shaded areas to reflect their separate estimation scheme (i.e., these effects were estimated while not controlling for further human demographic variables) as outlined in section 2.2.

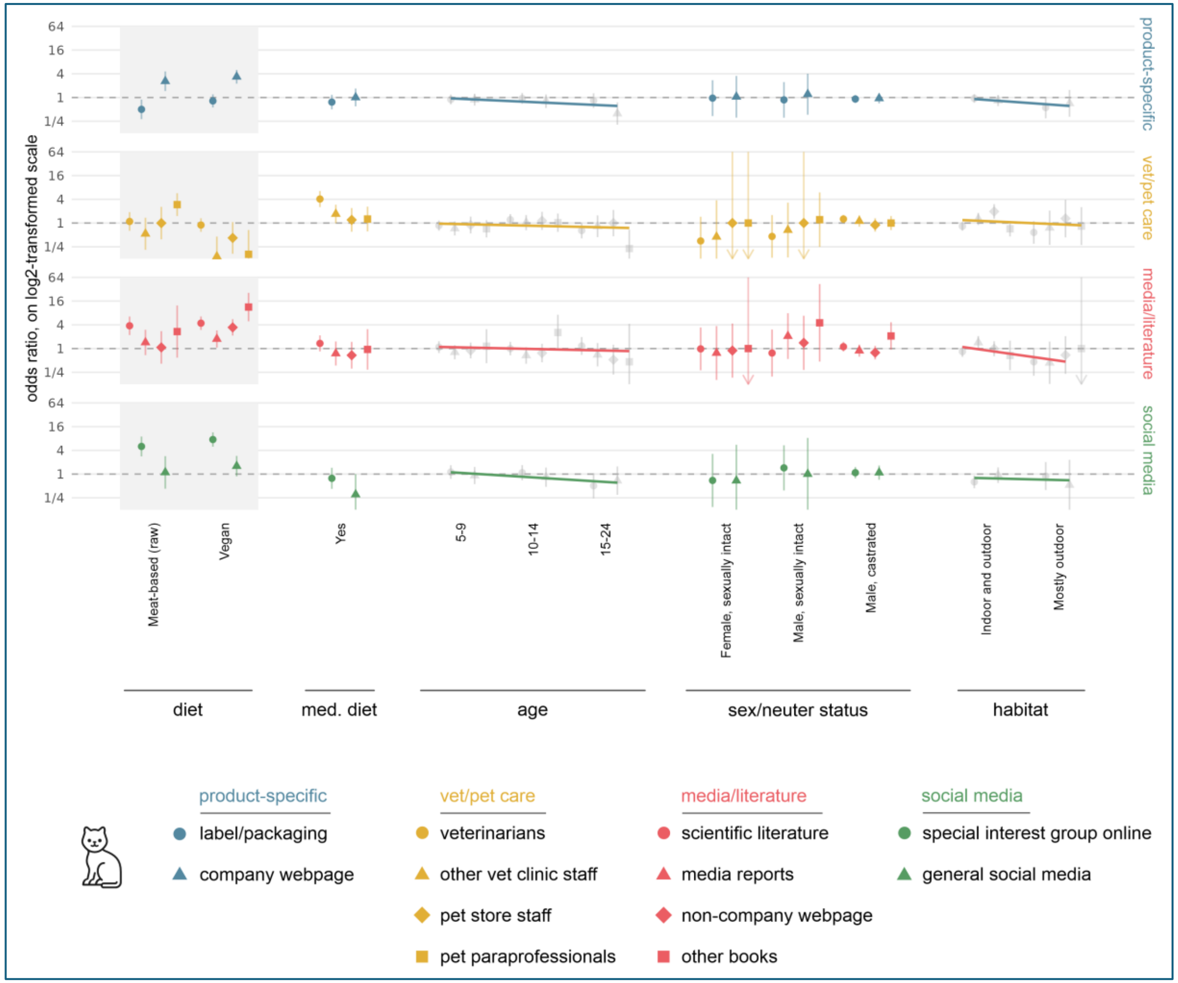

Figure S7. Logistic regression results on the associations between cat demographic characteristics and dietary information sources, relative to the reference characteristics for each group.

Note: This figure relates to Figure 13 from the main paper and illustrates the results regarding the individual items that comprise the right y-axis categories. The reference characteristics for these cats were: meat-based (conventional); no medical diet; aged 0–4; female, spayed; and mostly indoors. Effects are depicted as odds ratios, including 95% confidence intervals. Due to the exploratory character of this analysis, no multiple testing correction was applied and statistical significance was not investigated. Individual estimated effects of ordinal variables are grayed out in favor of an additional linear trend line. Effects of cat diet are highlighted through gray-shaded areas to reflect their separate estimation scheme (i.e., these effects were estimated while not controlling for further human demographic variables) as outlined in section 2.2.
